# Impaired Learning and Memory Ability Induced by a Bilaterally Hippocampal Injection of Streptozotocin in Mice: Involved With the Adaptive Changes of Synaptic Plasticity

**DOI:** 10.3389/fnagi.2021.633495

**Published:** 2021-03-01

**Authors:** Cong-Cong Qi, Xing-Xing Chen, Xin-Ran Gao, Jing-Xian Xu, Sen Liu, Jin-Fang Ge

**Affiliations:** ^1^Department of Laboratory Animal Science, Fudan University, Shanghai, China; ^2^School of Pharmacy, Anhui Medical University, Hefei, China; ^3^Anhui Provincial Laboratory of Inflammatory and Immunity Disease, Anhui Institute of Innovative Drugs, Hefei, China; ^4^The Key Laboratory of Anti-inflammatory and Immune Medicine, Ministry of Education, Anhui Medical University, Hefei, China

**Keywords:** Alzheimer disease, streptozotocin, BDNF, nesfatin-1 (NUCB2), NMDAR

## Abstract

**Background:** Alzheimer's disease (AD) is a neurodegenerative disease characterized by progressive cognitive decline, psychiatric symptoms and behavioral disorders, resulting in disability, and loss of self-sufficiency.

**Objective:** To establish an AD-like mice model, investigate the behavioral performance, and explore the potential mechanism.

**Methods:** Streptozotocin (STZ, 3 mg/kg) was microinjected bilaterally into the dorsal hippocampus of C57BL/6 mice, and the behavioral performance was observed. The serum concentrations of insulin and nesfatin-1 were measured by ELISA, and the activation of hippocampal microglia and astrocytes was assessed by immunohistochemistry. The protein expression of several molecular associated with the regulation of synaptic plasticity in the hippocampus and the pre-frontal cortex (PFC) was detected via western blotting.

**Results:** The STZ-microinjected model mice showed a slower bodyweight gain and higher serum concentration of insulin and nesfatin-1. Although there was no significant difference between groups with regard to the ability of balance and motor coordination, the model mice presented a decline of spontaneous movement and exploratory behavior, together with an impairment of learning and memory ability. Increased activated microglia was aggregated in the hippocampal dentate gyrus of model mice, together with an increase abundance of Aβ_1−42_ and Tau in the hippocampus and PFC. Moreover, the protein expression of NMDAR2A, NMDAR2B, SynGAP, PSD95, BDNF, and p-β-catenin/β-catenin were remarkably decreased in the hippocampus and the PFC of model mice, and the expression of p-GSK-3β (ser9)/GSK-3β were reduced in the hippocampus.

**Conclusion:** A bilateral hippocampal microinjection of STZ could induce not only AD-like behavioral performance in mice, but also adaptive changes of synaptic plasticity against neuroinflammatory and endocrinal injuries. The underlying mechanisms might be associated with the imbalanced expression of the key proteins of Wnt signaling pathway in the hippocampus and the PFC.

**Graphical Abstract:**
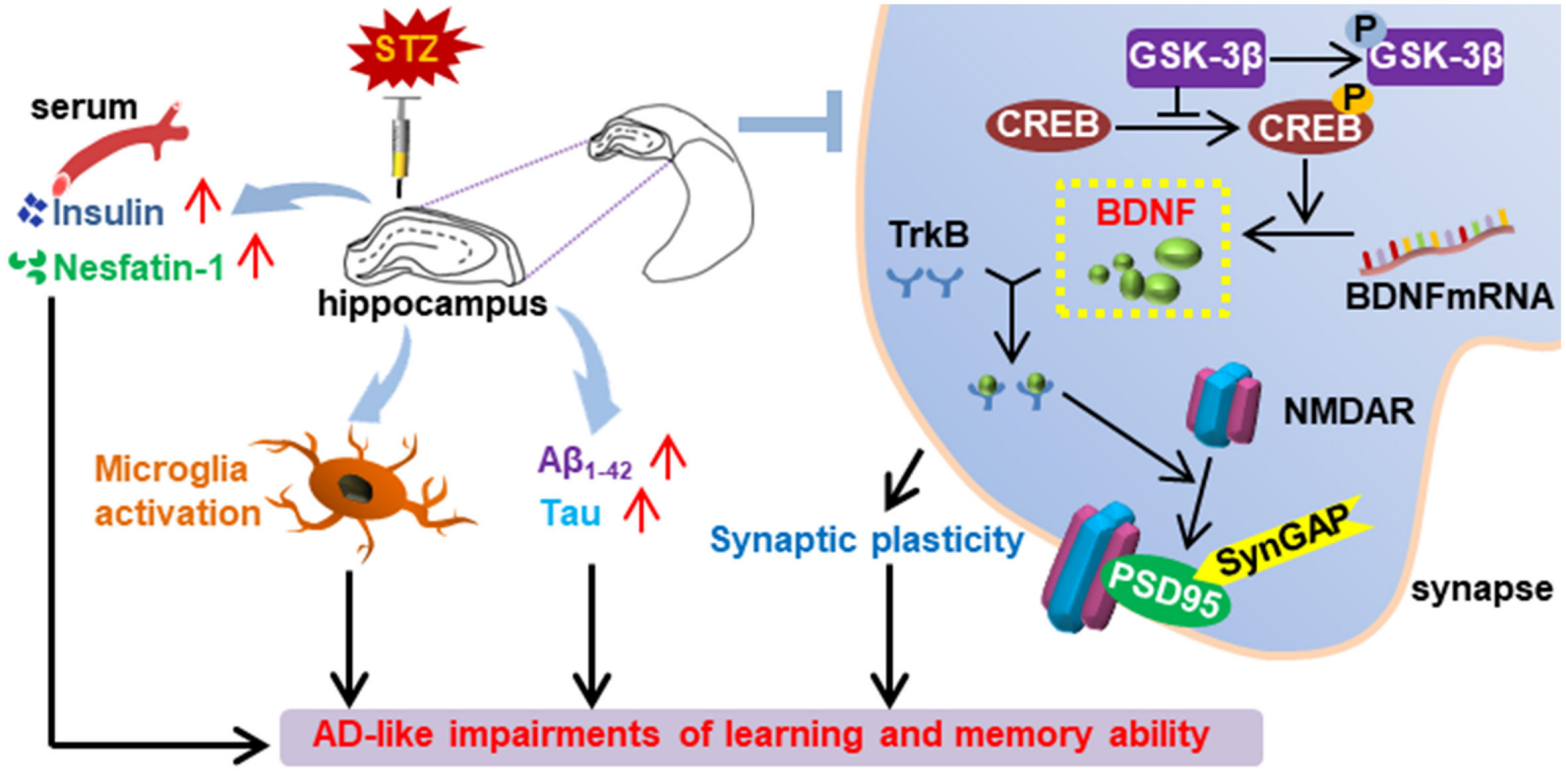
Summary of the effect of bilaterally hippocampal injection of streptozotocin on the mice.

## Introduction

Alzheimer's disease (AD) is a devastating neurodegenerative disorder characterized by a progressive deterioration of cognitive functions (Zameer et al., 2019). Targeted at increasing the activity of cholinergic neurons, cholinesterase inhibitors such as tacrine are clinically used in the treatment for AD with limited effect, but they could not slow or reverse the progress of AD. According to the classical amyloid cascade hypothesis, the deposition of amyloid beta (Aβ) is taken as the trigger of AD leading to the formation of senile plaques (SPs) and nerve fiber tangles (NFTs), resulting in the death of nerve cells and dementia eventually. However, the results of considerable clinical trials of anti-AD agents targeting at Aβ are mostly ended in failure (Hung and Fu, 2017). Although sodium oligomannate has received its first approval in 2019 in China for the treatment of mild to moderate AD (Wang et al., 2019; Syed, 2020), it is still one of the most imperative scientific issues to explore the pathogenesis of AD clearly, find potential therapeutic targets, and develop effective therapeutic agents in the field of neuropsychiatric diseases and public health.

Increasing evidence shows the important role of glutamate-mediated excitotoxity in the pathogenesis of AD, basing on which N-methyl-D-aspartate receptor (NMDARs/GluN) antagonist memantine has been approved by American Food and Drug Administration (FDA) in treatment for AD. Classical NMDAR is a heterotetramer composed of two GluN1 subunits and two GluN2 (A/B) combinations (Henneberger et al., 2013). It has been reported that GluN2A and GluN2B promote the induction and prolongation of synaptic plasticity in different ways (Brigman et al., 2010; Wang et al., 2011; Ballesteros et al., 2016). Declined NMDAR subunits are found in the hippocampus of AD patients, with a significant association with the abnormal presynaptic changes and cognitive deficits (Sze et al., 2001).

NMDARs are reported to be involved in the pathogenesis of AD via regulating the synaptic function (Muller et al., 2018). Synaptic Ras GTPase activation protein (SynGAP) located at excitatory synapses is reported to integrate with postsynaptic density protein 95 (PSD95), Shank and Ca^2+^/CaM kinase II (CaMKII) into key signaling protein complexes. The complex is responsible for the membrane binding between synaptic terminals and the neurotransmitter receptors including NMDARs and AMPAR in the postsynaptic density (Chen et al., 2015). These results suggest that NMDARs perhaps take part in the etiopathology of neuropsychiatric injury in AD, with different physiological and molecular characteristics for different subunits of NMDARs.

The canonical Wnt/β-catenin signaling pathway is required for the maintenance of normal brain structure and function (Gaesser and Fyffe-Maricich, 2016), and the deficit of Wnt/β-catenin pathway has been demonstrated in the brain of AD patients (Folke et al., 2019). Results of animal studies showed that blocking of the Wnt pathway could exacerbate the Tau hyper-phosphorylation, the formation and deposition of Aβ_1−42_, and the memory impairment (Tapia-Rojas and Inestrosa, 2018). Note that, abnormal function of Wnt signaling pathway can promote the production of Aβ, while Aβ, in turn, can inhibit the Wnt/β-catenin signaling pathway to aggravate the course of AD (Magdesian et al., 2008; Rodriguez et al., 2011; Liu et al., 2014).

The hippocampus and the pre-frontal cortex (PFC) are very important brain areas involving with the regulation of emotion and cognition (Hiser and Koenigs, 2018), and increasing data have demonstrated their structural and functional changes in AD patients or animal models (Teixeira et al., 2018; Zhong et al., 2018) (Jahanshahi et al., 2019). Given the important role of insulin resistance in the mechanism of both AD and type 2 diabetes mellitus, streptozotocin (STZ), a glucosamine-nitrosourea and DNA alkylating reagent that could result in insulin resistance, are used widely to establish animal models of AD or diabetes mellitus with multiple administration methods (Zameer et al., 2019). Results of animal studies showed that peripheral or central administration of STZ could result in not only insulin resistance, but also structural or functional dysfunction in hippocampus and PFC (Li et al., 2018; Mishra et al., 2018). Moreover, it has been reported that hippocampal injection of AAV-CD82 could induce AD-like cognitive deficits and impairments in neuronal spine density in young adult mice (Zhao et al., 2020). Targeted hippocampal overexpression of tumor necrosis factor-α-induced protein 8-like 2 (TIPE2) in APP/PS1 mice could result in an improvement of learning and memory, together with a reduced expression of inflammatory cytokines and increased expression of anti-inflammatory cytokines (Miao et al., 2019). These findings suggest that hippocampal targeted injection of stimulants or therapeutic drugs can be used effectively in research focusing on the pathogenesis of AD and evaluation of therapeutic effects.

It is a crucial experimental means and premise to establish effective animal models mimicking the pathophysiological process of human diseases. The main purpose of this study was to characterize the AD mice model via bilaterally hippocampal microinjection of STZ. The behavioral performance was observed via the sucrose preference test (SPT), rotarod test (RT), open field test (OFT), tail suspension test (TST), novel object recognition (NOR) test, Morris water maze (MWM), and contextual fear conditioning (CFC) test. The serum concentrations of insulin and nesfatin-1 were tested via enzyme-linked immunosorbent assay (ELISA). The activation of microglia and astrocytes and neurogenesis in the hippocampus was detected by immunofluorescent staining with ionized calcium-binding adapter molecule 1 (Iba-1), CD68, glial fibrillary acidic protein (GFAP), and doublecortin (DCX) respectively. The expression of Aβ_1−42_, Tau, synaptic plasticity related molecules, and key proteins of the Wnt pathway in the hippocampus and the pre-frontal cortex (PFC) were detected via western blot.

## Materials and Methods

### Animals

Twenty-six adult C57BL /6 male mice (bodyweight 25~30 g, aging 3 months) were purchased from and kept in the experimental animal center of Tongji University School of Medicine under the condition equipped with a commutative 12 h light/dark schedule. The mice were randomly divided into a control group (CON) and a STZ-injection model group (MOD) with 4–5 mice per cage and access to food and water *ad libitum*. The ambient temperature was maintained at 20 ± 2°C with 50 ± 5% relative humidity. All the animal care practices and experiment procedures were reviewed and approved by the Animal Experimentation Ethics Committee of Tongji University School of Medicine, in compliance with the National Institutes of Health Guide for the Care and Use of Laboratory Animals (NIH publication No.85-23, revised 1985).

### Stereotactic Intra-Hippocampal Microinjection of STZ and Experiment Design

Each mouse was anesthetized by intraperitoneal injection of 5% chloral hydrate (0.1 ml/10 g), secured to a platform placed in a stereotaxic apparatus (David Kopf; Tujunga, CA, USA) and the scalp incised. After the bregma was located, a rotary tool was used to drill into the skull and get two burr holes, according to the following sites of 2.1 mm posterior, 1.3 mm lateral, and 1.8 mm below. Mice in the MOD group received bilateral infusions of STZ (Sigma-Aldrich, s0130; 3 mg/kg) dissolved in 0.9% sterile saline into the hippocampus on both coordinates with a constant rate (500 nL/min) pulled by a micropipette puller. Correspondingly, mice in the CON group received bilateral infusions of 0.9% sterile saline. After the wound was closed, the animals were placed on the 37°C warming pad to recover until their respiration and locomotor activity returned to normal (Zhang et al., 2016).

**Figure 1:**
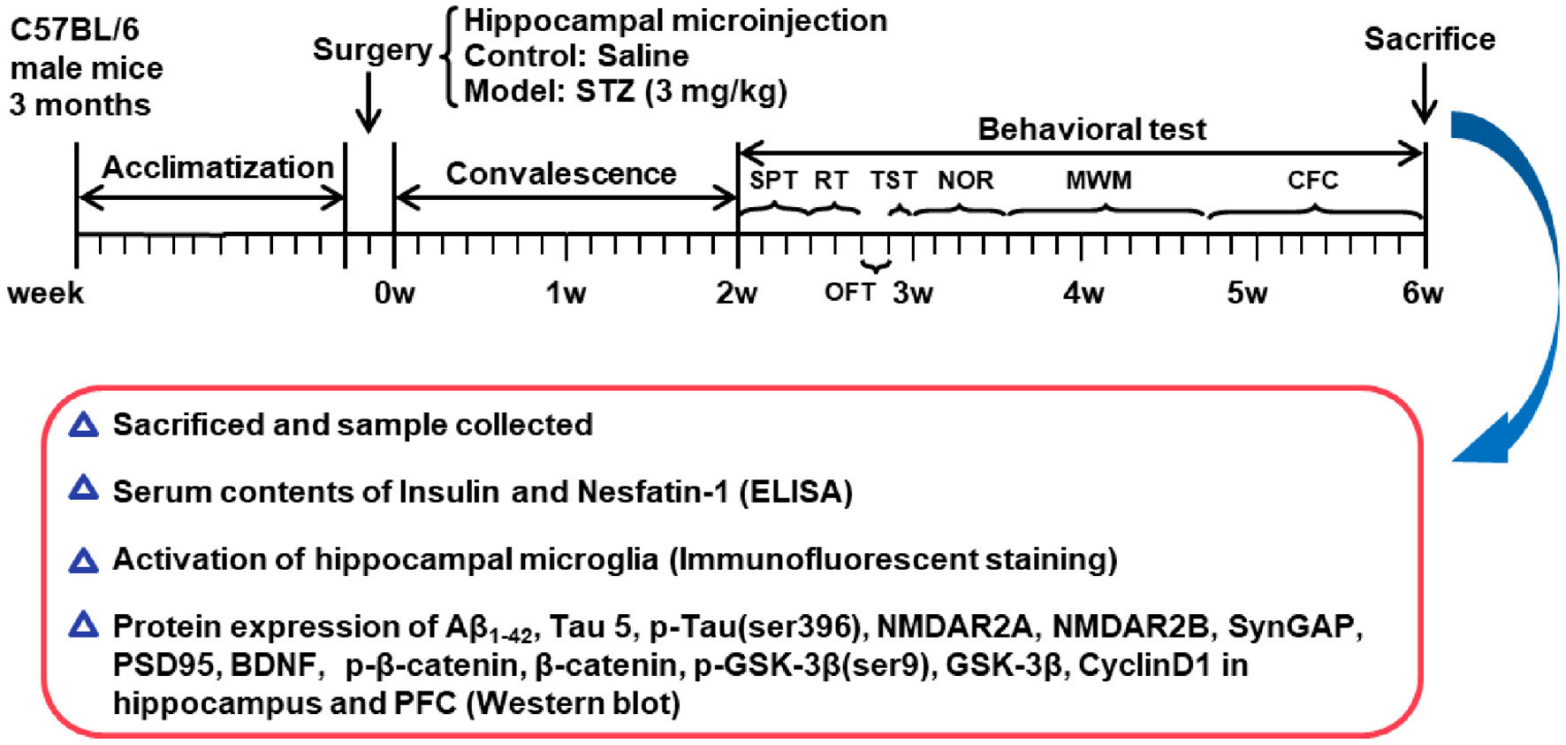
Schedule of the experimental design. STZ, streptozotocin; SPT, sucrose preference test; RT, rotarod test; OFT, open field test; TST, tail suspension test; NOR, novel object recognition test; MWM, Morris water maze; CFC, contextual fear conditioning test; BDNF, brain derived neurotrophic factor; PFC, pre-frontal cortex.

Bodyweight of mouse was monitored once a week. After 2 weeks of recovery from surgery, all mice were submitted to a chain of behavioral paradigms. The schedule of the experimental design is shown in Figure 1.

### Behavioral Test

All behavioral experiments were performed during the light phase of the light/dark cycle and in a sound-proof room with a neutral environment. All the mice in this study were given a 30-min habituation time after transported to the experimental rooms and subjected to the following behavioral tests. The experimenter was blind to the group identity of the tested mice. The behavioral tests were recorded with a camera and a trained researcher analyzed these videos.

The behavioral tests began with the SPT, followed in sequence by the RT, OFT, TST, NOR, MWM, and CFC test, which was shown in Figure 1.

#### Sucrose Preference Test (SPT)

The SPT was used to detect the anhedonia behavior in rodents (Ke et al., 2020). After a deprivation of food and water for 12 h, the mice were housed solely and had free access to two identical water bottles filled with either sterile water or 1% (w/v) sucrose solution, respectively. To eliminate the interference of the location factor, the positions of the bottles were swapped after 12 h. The mass of water and sucrose solution consumed were weighed after 12 and 24 h, respectively. The sucrose preference index (SPI) is defined as the ratio of the sucrose water ingested to the total amount of liquid intake.

#### Rotarod Test (RT)

Motor coordination and balance of mice was assessed by RT in a reliable way. The test was divided into two stages, namely the training period and the test period. The training period consisted of a uniform 10 rpm for 1 min, and an acceleration period of 4–40 rpm for 5 min with relaxing 30 min at least. The mice were placed on the rotating apparatus with their backs to the operator. Note that, once the mouse fell during the training, it will be put back immediately to make sure completion. The test period was conducted on the second day after the training by three trails on the rotating rod, and inter-trial interval was 1 h for each mouse. During the process of accelerating the rod device from 4 to 40 rpm over the course 5 min, the latency to fall off and the maximal rotation rate before the mouse fell down were recorded and evaluated (Kandimalla et al., 2018).

#### Open Field Test (OFT)

The OFT was conducted to synchronously explore the locomotion and exploration. The apparatus was made up of a square arena, with a white floor divided into 9 squares (10 cm × 10 cm) and enclosed by continuous 20.3 cm-high walls made of transparent plexiglass. Each mouse was put into it at one corner and headed toward the wall. The total distance traveled, latency to the central square, distance and duration in the central square, as well as the frequency through the central square over a 30 min period were recorded and analyzed by Activity Monitor software (Med Associates, St. Albans, VT, United States) (Zhang et al., 2019).

#### Tail Suspension Test (TST)

The TST was conducted as previously described (Zhou et al., 2016) with some modifications. Briefly, mice were individually suspended by tail (20 cm from floor) using a paper tape (1.5 cm from the tip of the end). Each mouse was suspended for a total of 6 min, and the duration of immobility was recorded during the final 4 min interval of the test. Mice were considered immobile only when they hung passively and completely motionless. In addition, the mouse that once climbed up their tails was not involved in this assay.

#### Novel Object Recognition (NOR) Test

The NOR test was performed to assess non-spatial memory in a 25 cm × 25 cm × 25 cm and black soundproof box (Zhu et al., 2014). The test consisted of three phase: habituation, training, and retention sessions. In the first stage, the single mouse was placed into the box facing the wall, having a preceding 10 min habituation once a day, for 3 consecutive days. During the training session, two indistinguishable objects were symmetrically fixed to the floor of the box, and each mouse was allowed to travel in the apparatus freely for 10 min. After the training, the mice were immediately returned to their home cages for 60 min at least. Ultimately, during the retention test, each mouse was placed back into the same box, with one of the familiar objects substituted by a novel object. Test mouse was then allowed to freely explore for 5 min and videotaped. New object recognition index, a ratio of the amount of time spent exploring the novel object over the total time spent exploring the both, was used to evaluate cognitive function. A mouse was scored as exploring the object when touching or sniffing the object with its nose or front legs, and facing the object at a distance within 1 cm (Wang D. et al., 2018).

#### Morris Water Maze (MWM) Test

MWM serves as a method of evaluating different aspects of spatial memory in rodents. The maze was equipped with a circular blue pool (diameter 120 cm, height 60 cm, filled with water to 40 cm high at 21°C), which was divided into four hypothetical, equal quadrants. A circular platform (11 cm diameter) was submerged approximately 1 cm below the surface of water in the center of the target quadrant. Several complex visual cues surrounded the pool diagonally and were used for facilitating orientation.

The test consisted of a 7-day acquisition phase and a 1-day probe test on the 8th day. During the acquisition phase, each mouse received 4 trials per day, and was trained to find the hidden platform. The mouse was placed into the pool facing the wall individually to find the submerged platform within 60 s, and stayed on it for 20 s. If the hidden-platform was not found within 60 s, the mouse was gently guided to the platform and held for 20 s. On the 8th day, the probe test was performed to determine memory retention, and the platform was removed. Each mouse was put into the pool from the diagonally opposite side of the previous platform, and permitted to swim for 60 s freely. The escape latency in the acquisition phase, total distance to the platform, the duration spent in and frequency to the target quadrant in the probe test were monitored and measured automatically by Noldus software (EthoVision XT 8.0, Noldus Technology) (Kandimalla et al., 2018).

#### Contextual Fear Conditioning (CFC) Test

The CFC paradigm was considered to assess the ability of associating a distinct context with aversive footshocks through hippocampus-dependent cognitive mechanisms in rodents as described previously (Dai et al., 2008). The mouse was subjected to a CFC test in a sound attenuating chamber (30 × 25 × 20 cm), the floor of which was consisted of parallel 2 mm diameter stainless steel rods spaced 8 mm apart. A preceding 10 min habituation was subjected to reduce the contribution of anxiety and stress on the outcome. 24 h later, mice were placed in the same chamber and allowed to explore freely for 120 s before receiving 8 unsignaled foot shocks (1.0 mA, 2 s) with inter-shock intervals of 60 s. Mice were immediately returned to their home cages 2 min after the final foot shock. The behavioral freezing to the context of mice was measured to evaluate contextual fear memory at three points: 1, 24, and 1 week after fear conditioning. The mice were placed back to the same context and scored freezing behavior for 660 s by the automated FreezeFrame system (Coulbourn Instruments), which digitizes the video signal at 4 Hz and compares movement frame by frame to score the amount of freezing. Freezing was defined as the absence of all movement except for that needed for breathing (Devi and Ohno, 2013).

### Measurement of the Serum Concentrations of Insulin and Nesfatin-1

Twenty-four hours after the last behavioral test, all mice were sacrificed and the blood was collected. The serum levels of insulin and nesfatin-1 were detected using commercially available enzyme-linked immunosorbent assay (ELISA) kits (insulin: Yuanye Biotech. Co., LTD, Shanghai, China; nesfatin-1: Cusabio Biotech. Co., LTD, Wuhan, China) according to the manufacturers' instructions.

### Immunofluorescent Staining

Three mice in each group were selected randomly and transcardially perfused with 0.01 M phosphate-buffered saline (PBS, pH 7.4) followed by 4% paraformaldehyde (PFA) in PBS. The whole brains were dissected out and post-fixed in 4% PFA overnight, followed by cryoprotection in 30% sucrose in PBS overnight. After embedded with optimum cutting temperature compound (OCT), the brains were cut into sagittal sections (25 m thick), and then stored at −80°C for immunostaining (Pruski et al., 2019).

Immunohistochemistry was performed as described previously (Zhu et al., 2014) with some modifications. Sections were re-hydrated washed in PBS for 3 × 10 min in a microwave followed by incubation with a blocking solution containing 5 % bovine serum albumin (BSA) and 0.1% Tween-20 in PBS (pH 7.4) for 6 min at 95°C to retrieve the antigen. Then, brain sections were incubated with rabbit anti-Iba-1 (1:1,000; LAK4357, Wako, Japan), rat anti-CD68 (1:200; MCA1957GA, Bio-Rad Company, United States), guinea pig anti-DCX (1:300; AB2253, Millipore, USA), or rabbit anti-GFAP (1:1,000; z0334, Dako, USA), at 4°C overnight. After several washes in PBS, sections were incubated with donkey anti-rabbit Alexa Fluor 488-conjugated IgG (1:500; R37118, Invitrogen, Carlsbad, CA, USA), and biotinylated horse anti-rat or horse anti-guinea pig IgG (1:500, Jackson ImmunoResearch, USA) at room temperature for 2 h. Ultimately, brain sections were incubated with streptavidin-Cy3 (1:1,000, 016160084, Jackson ImmunoResearch, West Grove, PA, USA) and counterstained with Hoechst 33258 (1:2,000, 94403, Sigma, St.Louis, MO, USA) at room temperature for 1 h. Fluorescent images were taken on a laser-scanning confocal fluorescent microscope (Leica TCS SP8, Leica Microsystems, Wetzlar, Germany).

### Western Blot Assays

Another 3 mice were selected randomly in each group, and sacrificed by cervical dislocation 24 h after the last behavioral test. The hippocampus and the PFC were dissected promptly, frozen in liquid nitrogen and stored at −80°C. The western blot protocol was carried out as described in our previous study. Briefly, the tissues were lysed in ice-cold radio immunoprecipitation assay (RIPA) buffer containing protease inhibitor cocktail (Roche, IN, USA) and the phosphatase inhibitor PhosSTOP (Roche, IN, USA). Determine equal amounts of proteins (20 μg) using the BCA Protein Assay Reagent Kit before loading onto the 12.5% SDS-PAGE. After separated from each other, the proteins were subsequently transferred onto the polyvinylidene difluoride membrane (PVDF). The membrane was blocked 2 h with 5% BSA in TBST at RT followed by incubation with primary antibodies respectively at 4°C overnight. The primary antibodies were as follows: anti-Aβ_1−42_ (1:1,000; 25524-1-AP; Proteintech), anti-Tau 5 (1:200; sc-58860; Santa Cruz), anti-p-Tau (ser396) (1:1,000; sc-32275; Santa Cruz), anti-NMDAR2A (1:2,000; 19953-1-AP; Proteintech), anti-NMDAR2B (1:2,000; 21920-1-AP; Proteintech), anti-SynGAP (1:2,000; 19739-1-AP; Proteintech), anti-PSD95 (1:2,000; MA1-045; Thermo), anti-BDNF (1:1,000; sc-546; Santa Cruz), anti-p-β-catenin (1:1,000; 9561; Cell Signaling Technology), anti-β-catenin (1:1,000; 9562; Cell Signaling Technology), anti-p-GSK-3β(ser9) (1:1,000; 9323; Cell Signaling Technology), anti-GSK-3β (1:1,000; 9315; Cell Signaling Technology), anti-CyclinD1 (1:1,000; ab190564; Abcam), and β-actin (1:2,000; TA-09; Zhongshan Biotechnology). Then the membranes were further incubated with a horseradish peroxidase-conjugated secondary antibody, goat anti-rabbit or goat anti-mouse secondary antibodies (Abcam), at RT for 1 h. All procedures were performed by rinsing in TBST for 10 min and repeated three times. After developed with the Easy Enhanced Chemiluminescence Western Blot Kit (Pierce Biotechnology, Rockford, IL, USA), the protein blots were visualized by a gel imaging system (Tanon Science and Technology Co., Ltd., Shanghai, China) and then images were processed and analyzed using Image J software (NIH) (Chen et al., 2019).

### Statistical Analysis

All statistical analyses were performed using SPSS (version 12.0.1, SPSS Inc., Chicago, IL, United States). The difference between groups was examined using Student's *t*-test. Data are expressed as means ± SEM and *P* < 0.05 was considered statistically significant. Correlation analysis was performed using a Pearson's correlation test.

## Results

### Bilaterally Hippocampal Injection of STZ Induced a Decreased Tendency of Body Weight-Gain

**Figure 2:**
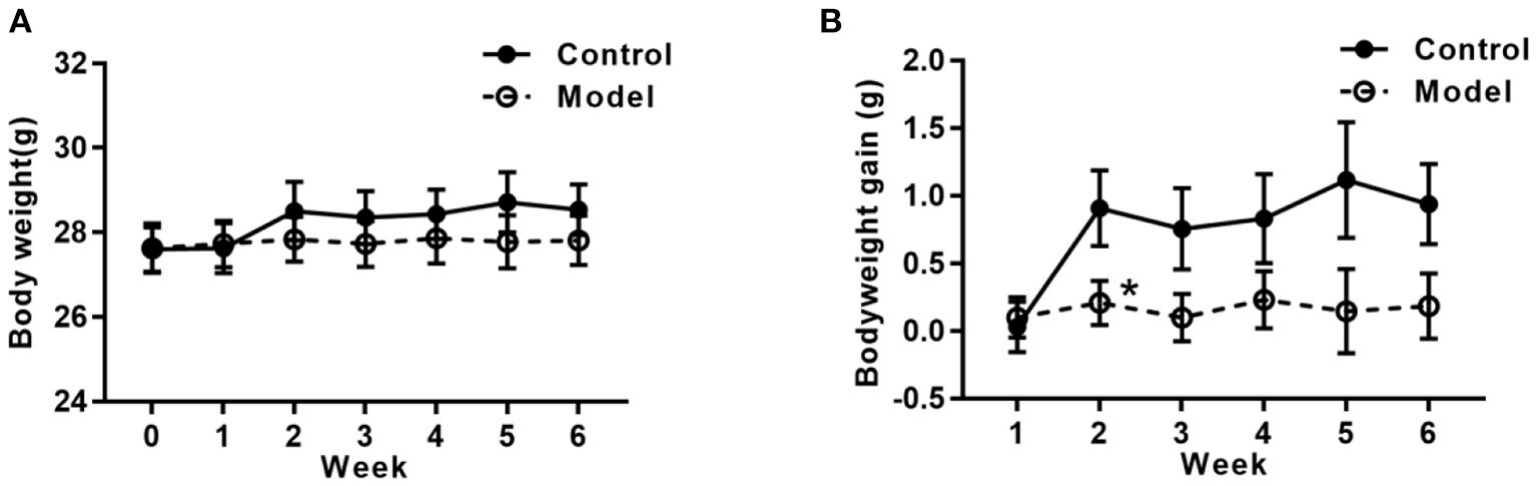
Effects of bilaterally hippocampal injection of STZ on the bodyweight gain in mice. The data are presented as the mean ± SEM, with 13 mice in each group. Although there was no significant difference between groups with regard to the bodyweight **(A)**, the net bodyweight gain **(B)** of mice injected by STZ in the model group was lower than that in the control group in the 2nd week. *P < 0.05, compared with the control group.

Figure 2 shows the bodyweight and bodyweight gain of mice in the two groups. As compared with that in the control group, the tendency of the bodyweight gain in the bilaterally hippocampal STZ-injected group was decreased, with a significant difference between groups during the 2nd week post-operation (*t* (24) =2.160, *P* = 0.041; Figure 2B).

### Bilaterally Hippocampal Injection of STZ Induced a Decrease of Locomotor Activity and Exploration Behavior in Mice in the OFT Without Significant Changes in the RT, TST, and SPT

**Figure 3:**
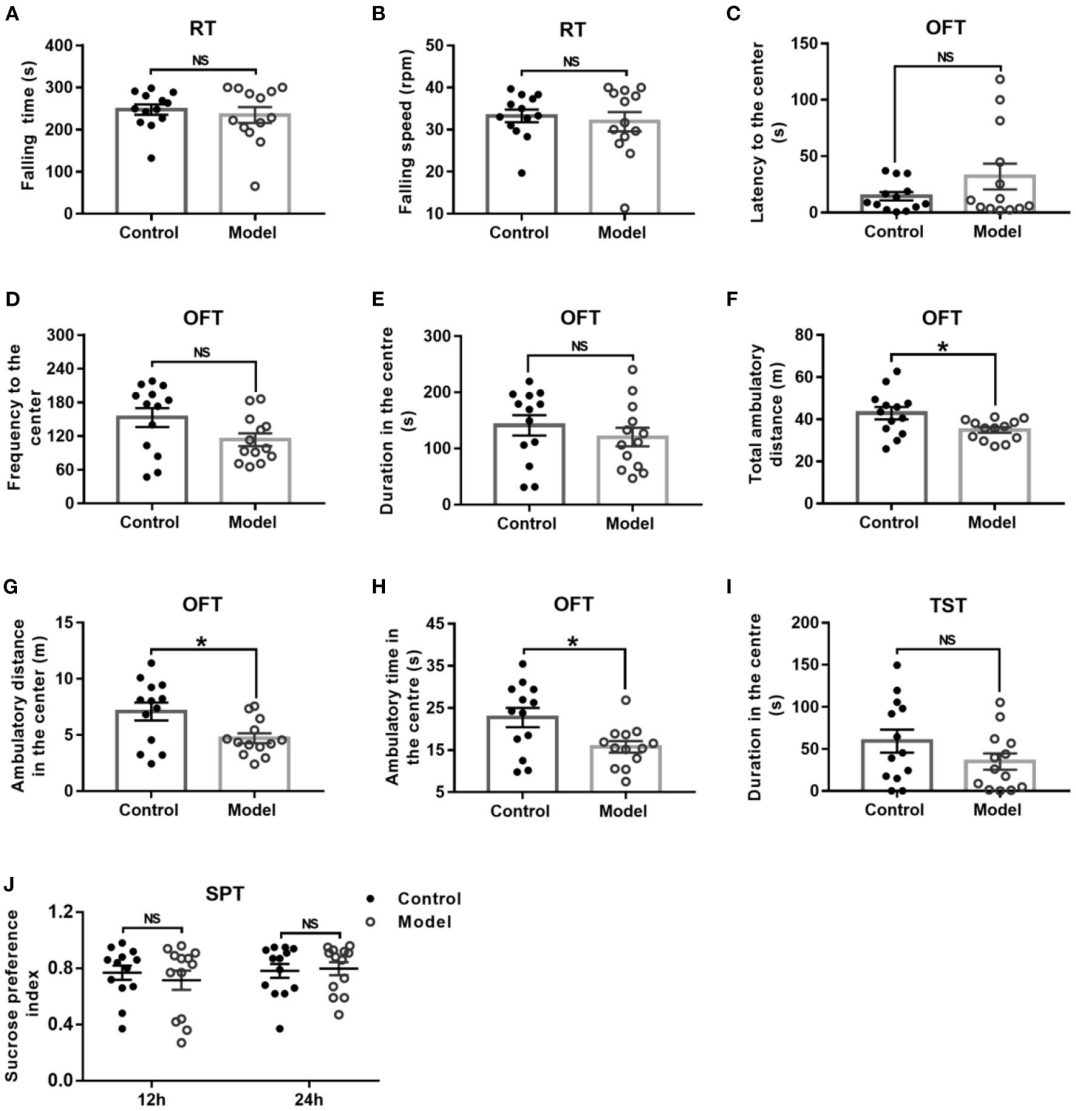
Effect of bilaterally hippocampal injection of STZ on the behavior performance of mice in the RT, OFT, TST, and SPT. The data are presented as the mean ± SEM, with 13 mice in each group. There was no significant difference between the two groups as regard to the falling time **(A)** and falling speed **(B)** in the RT. In the OFT, although there was no significant difference in the latency **(C)** and frequency **(D)** to the center, duration **(E)** in the center between the two groups, bilaterally hippocampal STZ-injected mice showed significant decreased total ambulatory distance traveled **(F)** in the area, as well as the ambulatory distance **(G)** and time **(H)** in the center zone than control mice. The immobility time **(I)** in the TST and the SPI **(J)** in the SPT are not remarkably different between groups. *P < 0.05; compared with the control group, NS is no significance.

General behavior performances of mice are shown in Figure 3, including the balance and motor coordination, automatic activity, exploratory and despair behavior, and anhedonia. As shown in Figures 3A,B, there was no significant difference between the two groups as regard to the falling time (*t* (24) = 0.561, *P* = 0.041; Figure 3A) and falling speed (*t* (24) = 0.496, *P* = 0.624; Figure 3B) in the RT. In the OFT, although there was no significant difference between groups with regards to the latency (*t* (24) = −1.439, *P* = 0.173; Figure 3C) and frequency (*t* (24) = −1.950, *P* = 0.063; Figure 3D) to the center, and duration in the center (*t* (24) = 0.856, *P* = 0.401; Figure 3E), the bilaterally hippocampal STZ-injected mice showed significantly decreased total ambulatory distance traveling in the area (*t* (24) = 2.474, *P* = 0.024; Figure 3F), as well as the ambulatory distance (*t* (24) =2.611, *P* = 0.017; Figure 3G) and ambulatory time (*t* (24) = 2.598, *P* = 0.017; Figure 3H) in the center zone as compared with the control ones. The immobility time in the TST (*t* (24) = 1.445, *P* = 0.161; Figure 3I) and the sucrose preference index in the SPT (12 h, *t* (24) = −0.257, *P* = 0.799; 24 h, *t* (24) = −0.239, *P* = 0.813, Figure 3J) were not remarkably different between groups.

### Bilaterally Hippocampal Injection of STZ Induced an Impairment of Learning and Memory Ability in Mice in the NOR and MWM, but Not CFC

**Figure 4:**
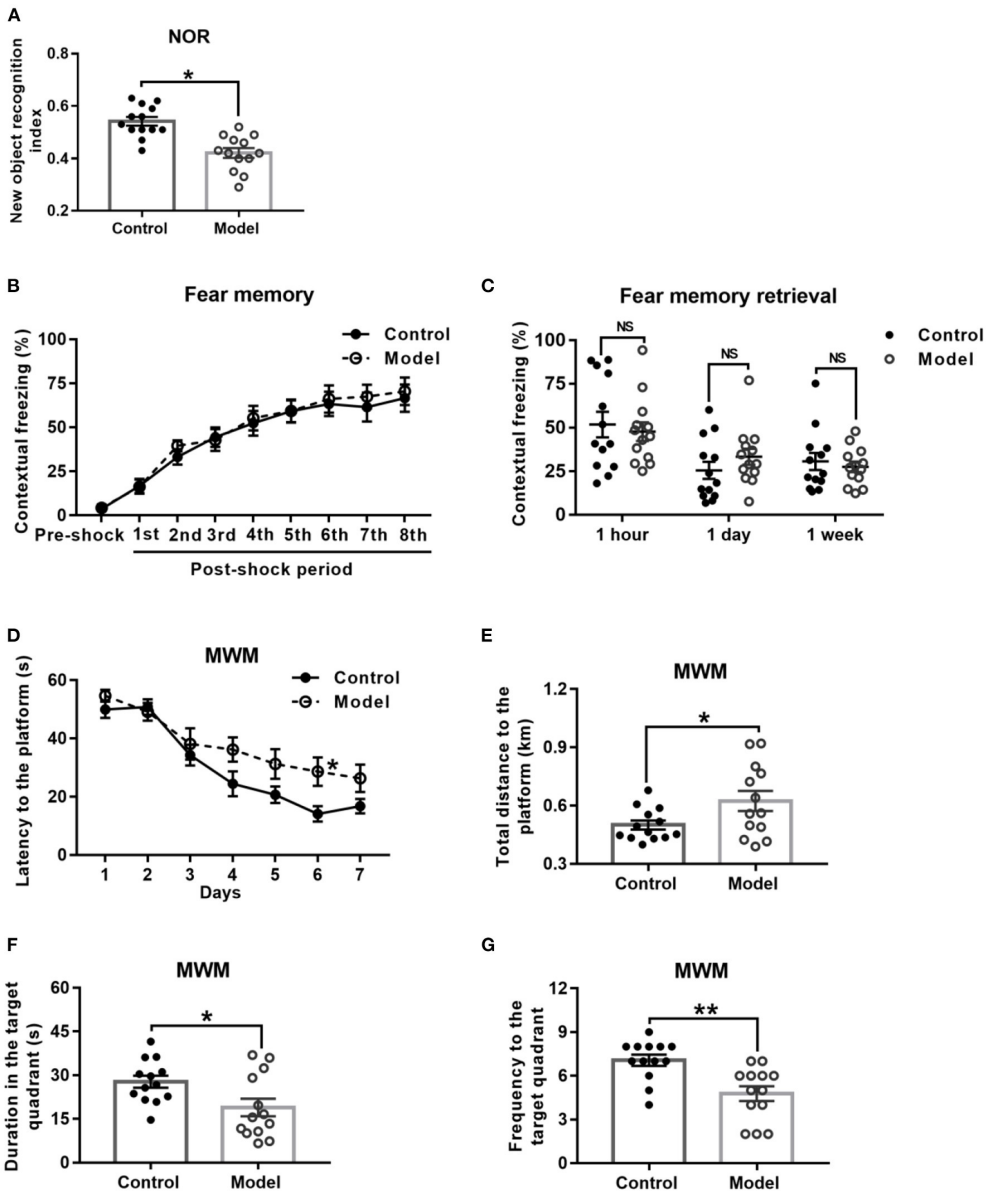
Effect of bilaterally hippocampal injection of STZ on the behavior performance of mice in the NOR, CFC and MWM. The data are presented as the mean ± SEM, with 13 mice in each group. In the NOR, compared with control mice, the novel object recognition index of model mice **(A)** was remarkably reduced. There was no significant difference between the two groups in the stage of fear memory formation **(B)** and freezing time of mice when the fear memory was extracted after 1 h, 1 day, and 1 week **(C)** in the CFC. In the MWM, during the acquisition phase, bilaterally hippocampal STZ-injected mice took longer time to find the submerged platform than the control mice did on 6th day **(D)**. During the probe trial, the model mice showed increased total swimming distance to the platform **(E)**, decreased duration **(F)** and less frequency **(G)** in the target quadrant. *P < 0.05, **P < 0.01; compared with the control group, NS is no significance.

Behavioral performance of mice in the NOR task is shown in Figure 4A. As compared with the control mice, the bilaterally hippocampal STZ-injected mice spent less time exploring the novel object, with a significant decrease of the novel object recognition index (*t* (24) = 2.091, *P* = 0.047; Figure 4A).

Figure 4B shows the performance of mice in the stage of fear memory formation in the CFC test, and there was no significant difference between the two groups. Moreover, the amount of freezing to context (Figure 4C) of the bilaterally hippocampal STZ-injected mice was not observably different from that of control ones, when the fear memories were extracted in the same environment 1 h, 1 and 7 days after formation of fear memory (1 h, *t* (24) = 0.448, *P* = 0.658; 1 day, *t* (24) = −1.158, *P* = 0.258; 7 days, *t* (24) = 0.542, *P* = 0.592, Figure 4C).

The performance of mice during the acquisition phase in the MWM is shown in Figure 4D. All the mice in this study learned how to locate the platform, as revealed by the sharp decline in the escape latency to the submerged platform. Moreover, the bilaterally hippocampal STZ-injected mice spent a longer time to find the escape platform than the control ones did on the sixth day (*t* (24) = −2.611, *P* = 0.017; Figure 4D). In the probe test, as compared with that of the control group, the total swimming distance to the platform (*t* (24) = −2.179, *P* = 0.044; Figure 4E) of the model mice was significantly extended, together with a shorter duration (*t* (24) = 2.411, *P* = 0.024; Figure 4F) in and less the frequency (*t* (24) = 3.625, *P* = 0.001; Figure 4G) to the target quadrant.

### Bilaterally Hippocampal Injection of STZ Increased the Serum Concentrations of Insulin and Nesfatin-1 in Mice

**Figure 5:**
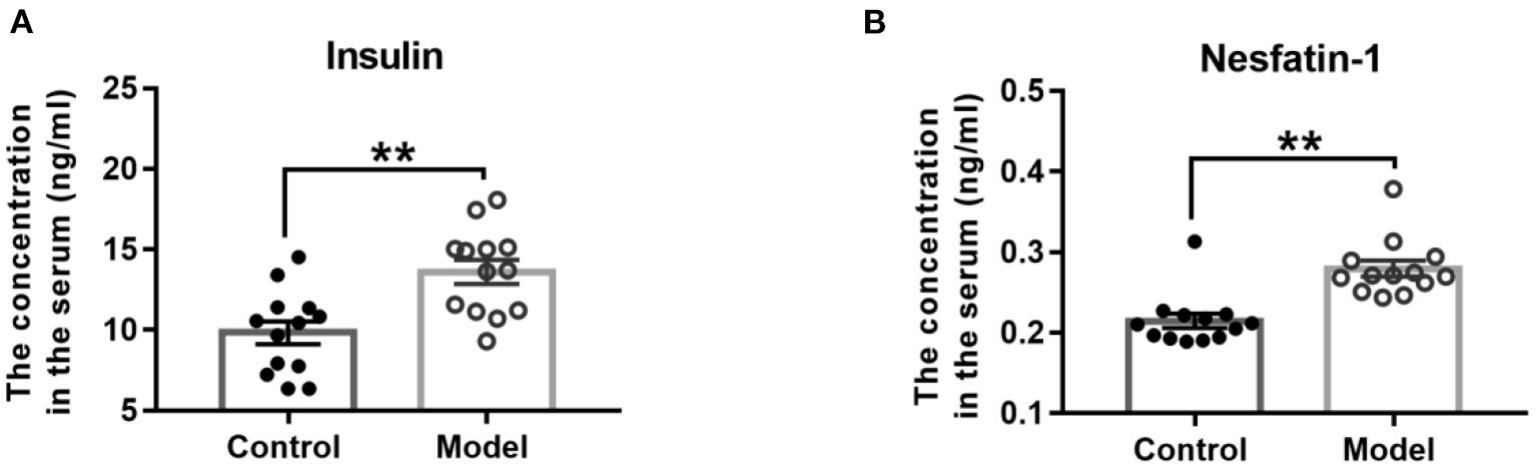
Effect of bilaterally hippocampal injection of STZ on the serum concentrations of insulin and nesfatin-1. The data are presented as the mean ± SEM, with 13 mice in each group. Compared with those in the control mice, the serum concentrations of insulin **(A)** and nesfatin-1 **(B)** were observably increased in the bilaterally hippocampal STZ-injected mice. **P < 0.01, compared with the control group.

As Figure 5 shown, compared with those in the control mice, the serum concentrations of insulin (*t* (24) = −3.665, *P* = 0.001; Figure 5A) and nesfatin-1 (*t* (24) = −4.850, *P* < 0.001; Figure 5B) were observably increased in the bilaterally hippocampal STZ-injected mice.

### Bilaterally Hippocampal Injection of STZ Promoted Microglia to Aggregate and Activate in the Hippocampus of Mice

**Figure 6:**
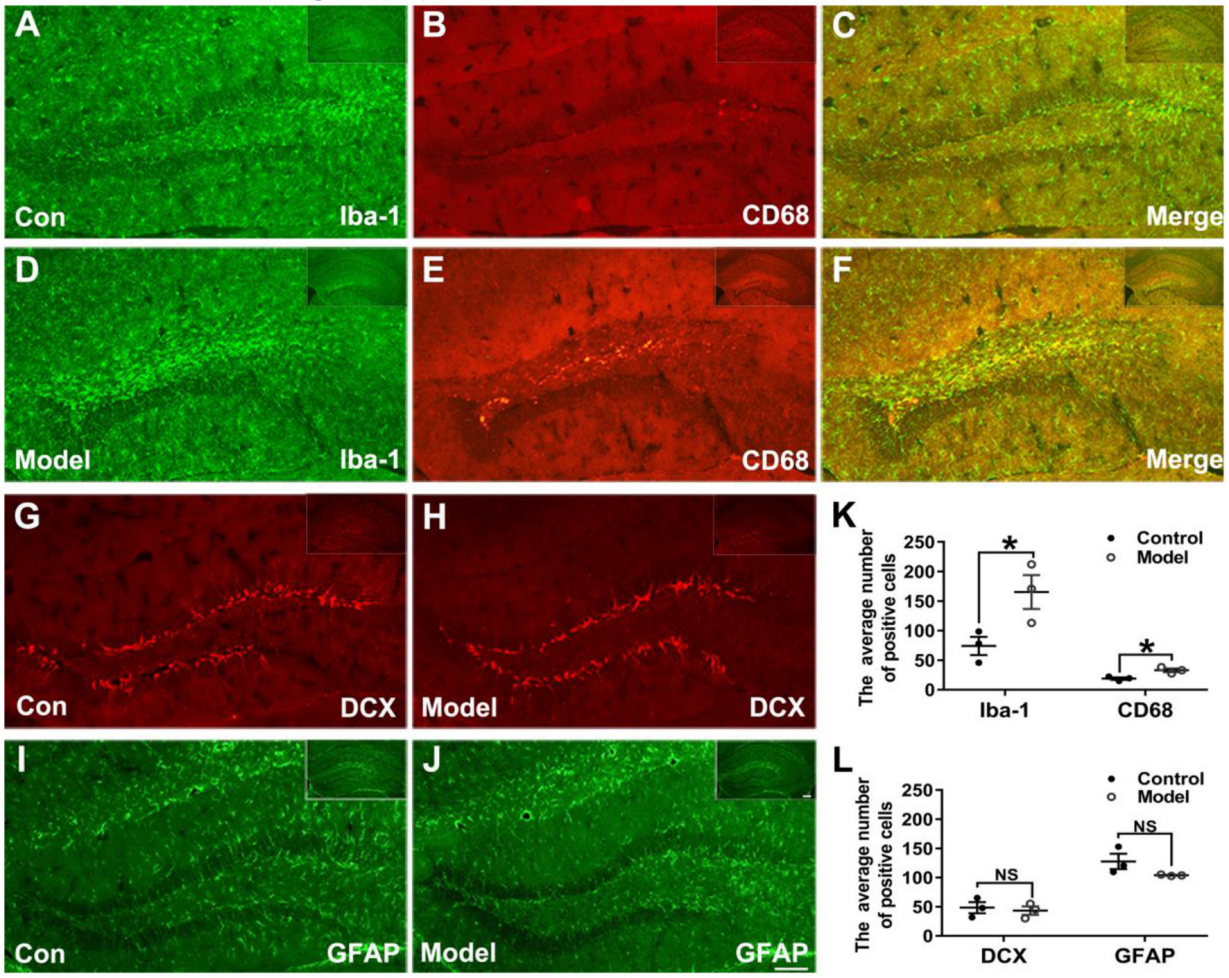
Effects of bilaterally hippocampal injection of STZ on the activation of microglias in the dentate gyrus of hippocampus of mice. **(A–F)** shows immunofluorescent microscopy of Iba-1 (green) and CD68 (red) in hippocampus tissues and the dentate gyrus. **(G–J)** shows immunofluorescent microscopy of DCX (red) and GFAP (green) in hippocampus tissues and the dentate gyrus (DG). Compared with those in the control mice, the number of Iba-1 **(A,D)** and CD68 **(B,E)** positive microglia ascends significantly in the DG tissues of the model group **(K)**. There was no significant difference in the number of positive cells of DCX **(G,H)** and GFAP **(I,J)** between the two groups. The data in **(K)** and **(L)** are presented as the mean ± SEM, with 3 mice in each group. Scale bars = 100 μm in J (applies to **A–J**), 200 μm in insets. *P < 0.05; compared with the control group, NS is no significance.

Figures 6A–F shows the typical immunofluorescent images of Iba-1 (green) and CD68 (red) in the mice hippocampal sections. As shown in Figures 6A,D, the Iba-1 (green) positive microglia clustered at the dentate gyrus (DG) of the bilaterally hippocampal STZ-injected mice, while such a phenomenon was not detected in the control group. Interestingly, as shown in Figure 6K, a dramatic ascending number of positive Iba-1 (green) marked microglia (*t* (4) = −2.796, *P* = 0.049; Figure 6K) and CD68 (red) marked activated microglia (*t* (4) = −3.806, *P* = 0.019; Figure 6K) were presented in the DG of the hippocampal tissues of model mice, and the activated microglial marker CD68 was mostly clustered around the hippocampal injection site.

Figures 6G–J shows the typical immunofluorescent images of DCX (red) and GFAP (green) in the mice brain sections. There was no significant difference between groups as regard to the number of DCX (red) positive cells (*t* (4) = 0.417, *P* = 0.698; Figures 6G,H,L). As shown in the Figures 6I,J,L, the GFAP (green) positive cells in the mice brain of the two groups were uniformly distributed in the hippocampus (*t* (4) =1.814, *P* = 0.210; Figure 6L).

### Bilaterally Hippocampal Injection of STZ Induced an Increased Expression of Aβ_1−42_ and Both the Total and Phosphorylated Tau in the Hippocampus and the PFC in Mice

**Figure 7:**
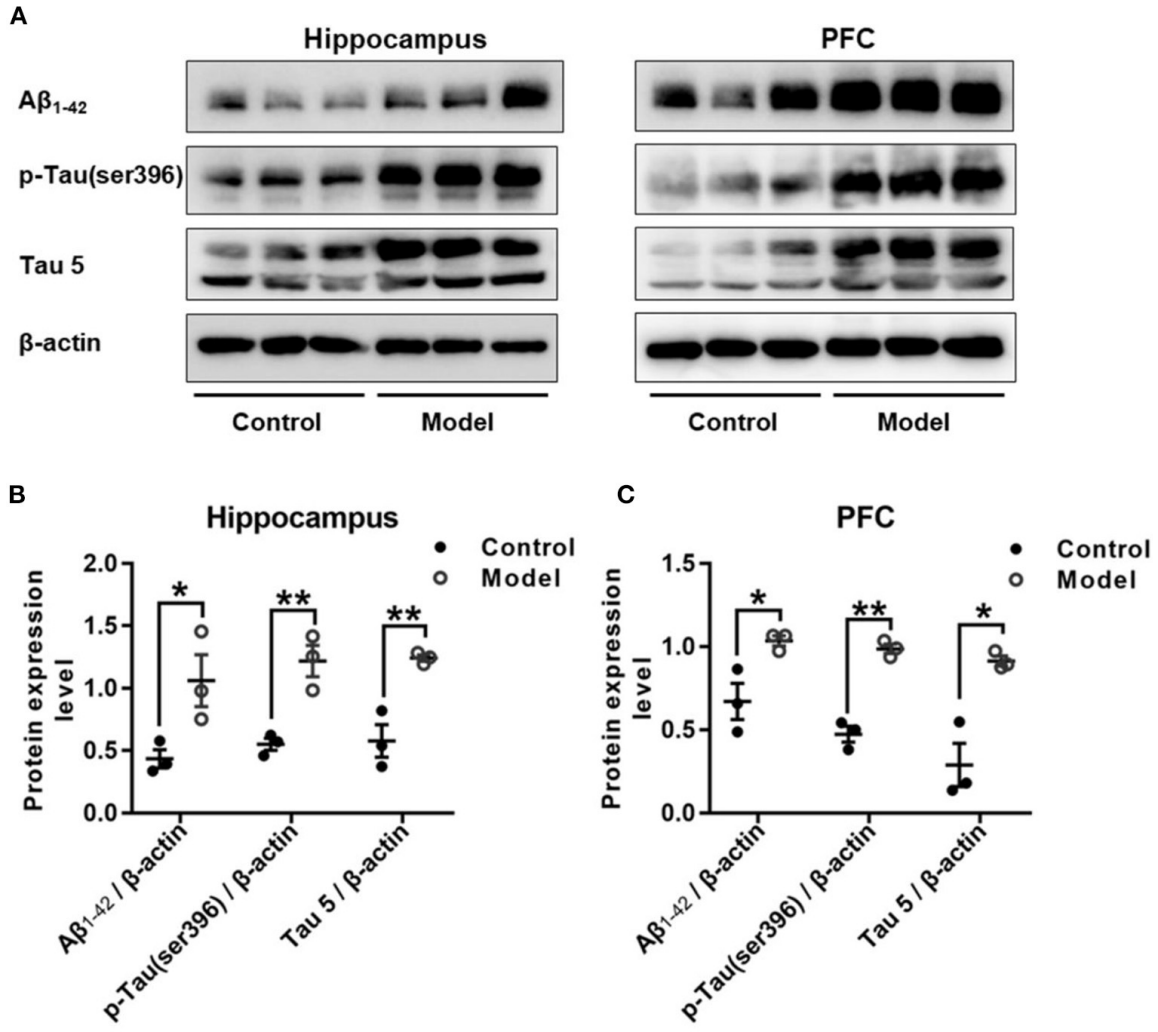
Effect of bilaterally hippocampal injection of STZ on amyloid beta and Tau protein in the hippocampus and PFC of model mice. Typical graph **(A)** and statistical analyses **(B,C)** of the western blot results. Data in **(B,C)** are presented as the mean ± SEM (*n* = 3 for each group). Compared with that in the control mice, the relative expression of Aβ_1−42_, p-Tau (ser396) and Tau 5 was increased in the hippocampus **(A,B)** and the PFC **(A,C)** of the model mice. *P < 0.05, **P < 0.01; compared with the control group.

Figure 7 shows the expression of Aβ_1−42_, p-Tau (ser396) and Tau 5 in the hippocampus and PFC of the mice. As compared with that in the control group, the relative expression of Aβ_1−42_ was remarkably increased in the hippocampus (*t* (4) = 2.947, *P* = 0.046; Figure 7B) and PFC (*t* (4) = 2.175, *P* = 0.032; Figure 7C), and both the expression level of p-Tau (ser396) and Tau 5 were significant increase in the hippocampus (*t* (4) = 2.195, *P* = 0.008; Figure 7B; *t* (4) = 4.092, *P* = 0.007; Figure 7B) and the PFC (*t* (4) = 1.389, *P* = 0.001; Figure 7C; *t* (4) = 8.196, *P* = 0.035; Figure 7C).

### Bilaterally Hippocampal Injection of STZ Induced an Imbalanced Expression of Synaptic Plasticity Related Proteins in the Hippocampus and the PFC in Mice

**Figure 8:**
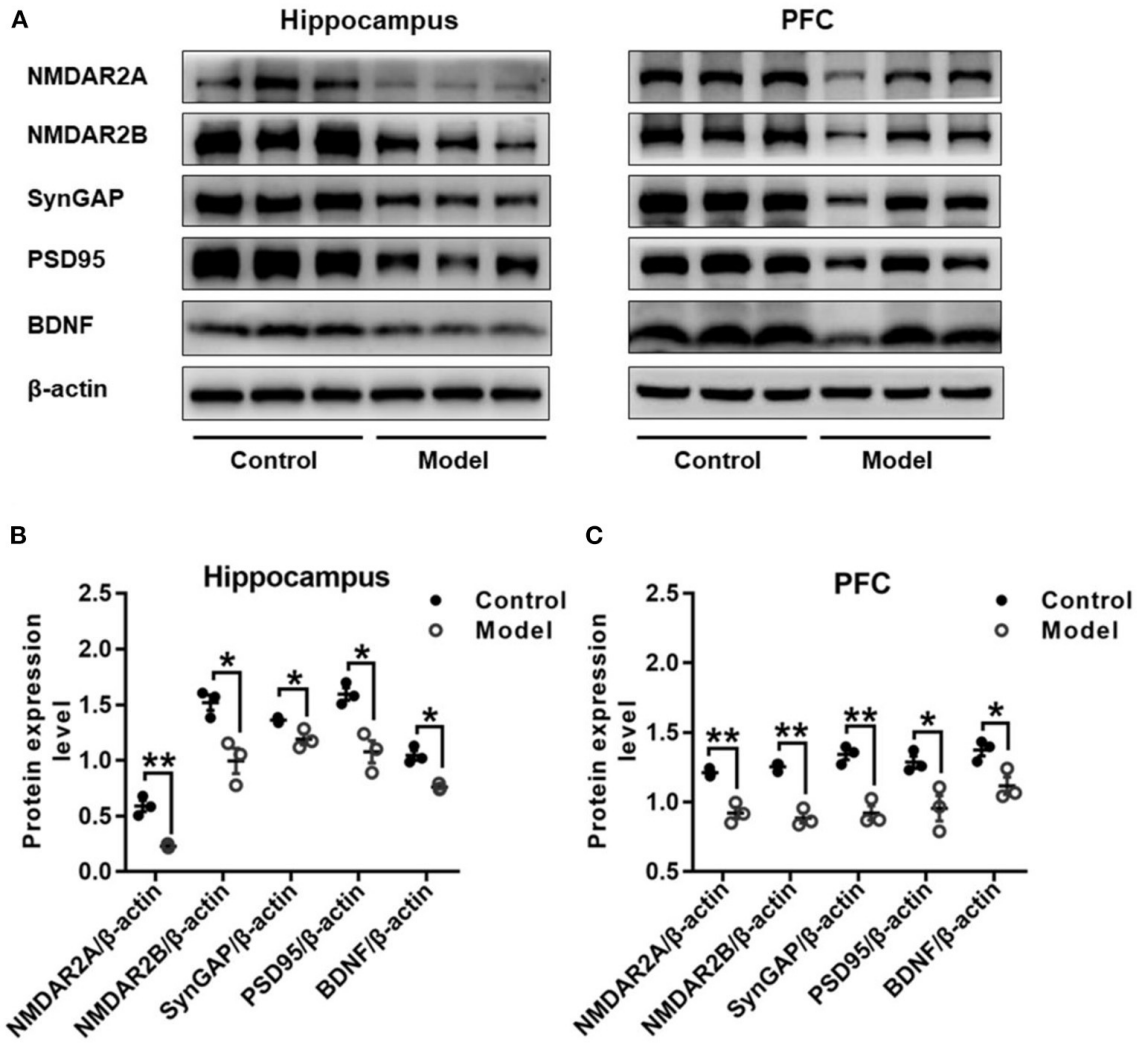
Effects of bilaterally hippocampal injection of STZ on the expression of synaptic plasticity related proteins in the hippocampus and PFC of model mice. Typical graph **(A)** and statistical analyses **(B,C)** of the western blot results. Data in **(B,C)** are presented as the mean ± SEM (*n* = 3 for each group). In the hippocampus **(A,B)**, the expression of NMDAR2A, NMDAR2B, SynGAP, PSD95, and BDNF was all decreased in the model group. The proteins expression situation in PFC is similar to that in hippocampus **(A,C)**. *P < 0.05, **P < 0.01; compared with the control group.

Figure 8 shows the protein expressions of NMDAR2A, NMDAR2B, SynGAP, PSD95, and BDNF in the hippocampus and PFC of mice. Compared with those in the control mice, the expression levels of NMDAR2A (Hippocampus: *t* (4) = 7.037, *P* = 0.002, Figure 8B; PFC: *t* (4) = 6.490, *P* = 0.003, Figure 8C), NMDAR2B (Hippocampus: *t* (4) = 3.962, *P* = 0.017, Figure 8B; PFC: *t* (4) = 9.076, *P* = 0.001, Figure 8C), SynGAP (Hippocampus: *t* (4) = 3.391, *P* = 0.028, Figure 8B; PFC: *t* (4) = 6.613, *P* = 0.003, Figure 8C), PSD95 (Hippocampus: *t* (4) = 4.461, *P* = 0.011, Figure 8B; PFC: *t* (4) = 3.280, *P* = 0.030, Figure 8C) and BDNF (Hippocampus: *t* (4) = 6.092, *P* = 0.004, Figure 8B; PFC: *t* (4) = 3.423, *P* = 0.027, Figure 8C) were remarkably decreased in both the hippocampus and the PFC of bilaterally hippocampal STZ-injected mice. Despite the small sample size, significant positive correlations were observed between the protein expression of BDNF and NMDAR2A (Hippocampus: *r* = 0.996, *P* < 0.001, Supplementary Figure 1A; PFC: *r* = 0.960, *P* = 0.002, Supplementary Figure 1B), SynGAP (Hippocampus: *r* = 0.850, *P* = 0.032, Supplementary Figure 1C; PFC: *r* = 0.880, *P* = 0.021, Supplementary Figure 1D), or PSD95 (Hippocampus: *r* = 0.857, *P* = 0.029, Supplementary Figure 1E; PFC: *r* = 0.894, *P* = 0.016, Supplementary Figure 1F). Moreover, significant positively correlations were also observed between the protein expression of NMDAR2A and NMDAR2B (Hippocampus: *r* = 0.820, *P* = 0.045, Supplementary Figure 2A; PFC: *r* = 0.985, *P* < 0.001, Supplementary Figure 2B), SynGAP (Hippocampus: *r* = 0.848, *P* = 0.033, Supplementary Figure 2C; PFC: *r* = 0.952, *P* = 0.003, Supplementary Figure 2D), or PSD95 (Hippocampus: *r* = 0.851, *P* = 0.032, Supplementary Figure 2E; PFC: *r* = 0.871, *P* = 0.024, Supplementary Figure 2F).

### Bilaterally Hippocampal Injection of STZ Induced a Dysregulation of Wnt/β-catenin Signaling Pathway in the Hippocampus and the PFC in Mice

**Figure 9:**
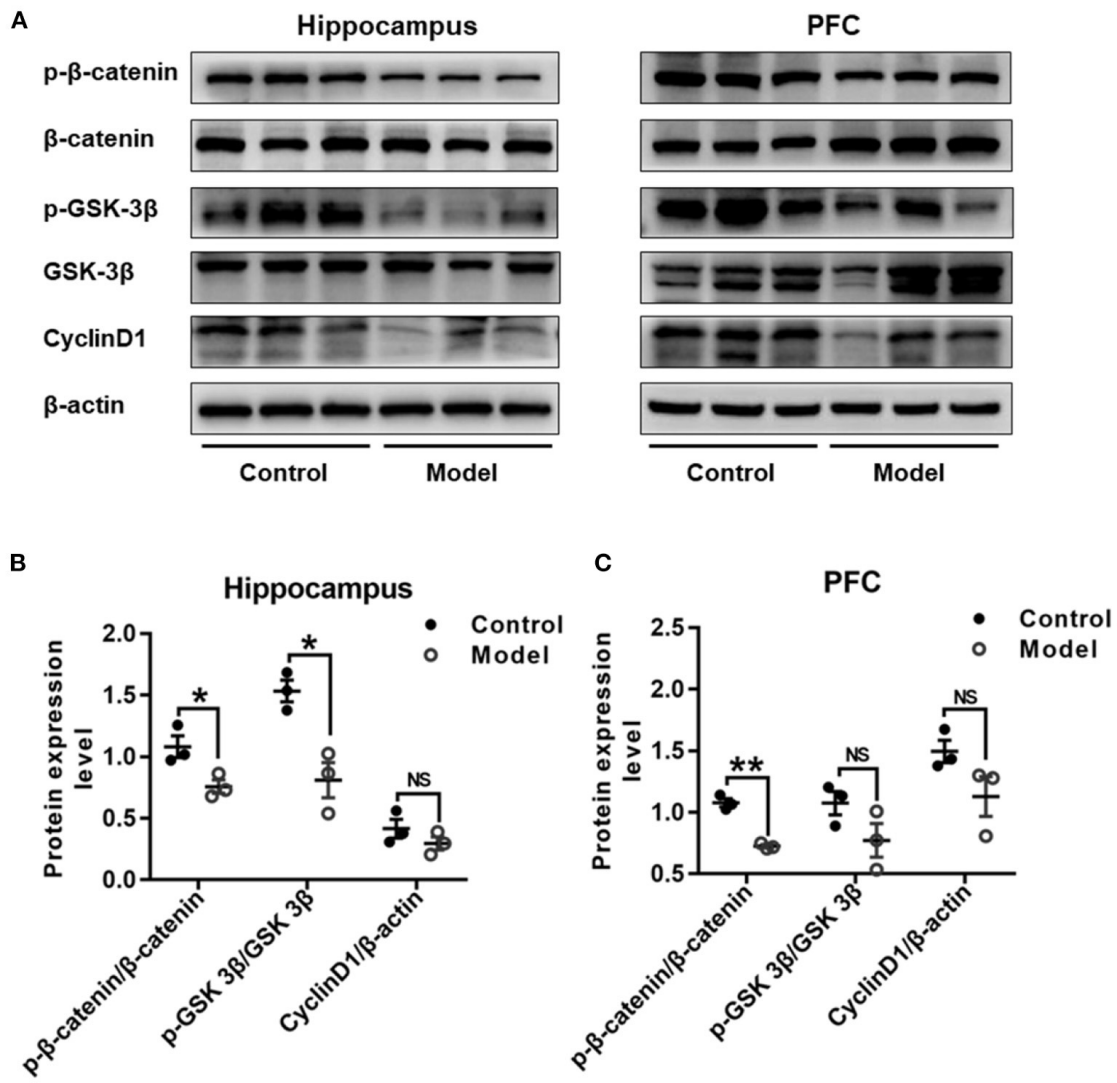
Effect of bilaterally hippocampal injection of STZ on the key protein expression of Wnt/β-catenin pathway in the hippocampus and prefrontal cortex of model mice. Typical graph **(A)** and statistical analyses **(B,C)** of the western blot results. Data in **(B,C)** are presented as the mean ± SEM (*n* = 3 for each group). In the hippocampus **(A,B)**, the expression of p-β-catenin/β-catenin and p-GSK-3β(ser9)/GSK-3β was decreased in the model group, without CyclinD1. In PFC **(A,C)**, although the expression of p-β-catenin/β-catenin of model mice was lesser than that of control mice, but not p-GSK-3β (ser9)/GSK-3β and CyclinD1. *P < 0.05, **P < 0.01; compared with the control group, NS is no significance.

Figure 9 shows the expression of key proteins in the Wnt/β-catenin pathway in the hippocampus and PFC of the mice. As compared with that in the control group, the relative expression of p-β-catenin/β-catenin in both the hippocampus (*t* (4) =3.111, *P* = 0.036; Figure 9B) and the PFC (*t* (4) = 9.748, *P* = 0.001; Figure 9C) were remarkably declined in bilaterally hippocampal STZ-injected mice, and the expression of p-GSK-3β (ser9)/GSK-3β was decreased in the hippocampus (*t* (4) = 4.306, *P* = 0.013; Figure 9B), but not the PFC (*t* (4) = 1.821, *P* = 0.143; Figure 9C). No significant changes were found between groups as regard to the protein expression of CyclinD1 in both the hippocampus (*t* (4) = 1.284, *P* = 0.269; Figure 9B) and the PFC (*t* (4) = 1.988, *P* = 0.118; Figure 9C). The results of Pearson's correlation analysis showed that the protein expression of p-GSK-3β/GSK-3β was positively correlated with BDNF (*r* = 0.939, *P* = 0.005; Supplementary Figure 3A), NMDAR2A (*r* =0.937, *P* = 0.006; Supplementary Figure 3B), or synGAP (*r* = 0.960, *P* = 0.002; Supplementary Figure 3C) in the hippocampus.

## Discussion

In the present study, an AD mouse model was established via bilaterally hippocampal injection of STZ. The results showed that a bilaterally hippocampal injection of STZ could induce a decreased tendency of bodyweight gain in mice, together with remarkably increased serum concentrations of insulin and nesfatin-1. Results of behavioral tests demonstrated that the spontaneous movement and exploratory behavior were decreased in the bilaterally hippocampal STZ-injected mice as compared with the control ones. More importantly, the model mice showed a task-specific impairment of learning and memory, as indicated by the declined novel object recognition index in the NOR, and the longer escape latency and swimming distance in the acquisition of MWM, decreased duration and less frequency in the target quadrant during the navigation phase of MWW. Moreover, our results showed that the microglia was aggregated around the injection site, and the number of activated microglia increased remarkably in the DG of the model mice. Furthermore, apart from the increased abundance of Aβ_1−42_ and Tau in the hippocampus and PFC of the model mice, the expression of NMDAR2A, NMDAR2B, SynGAP, PSD95, BDNF, and phosphorylated β-catenin were remarkably reduced in the hippocampus and the PFC, and the phosphorylation of GSK-3β was declined in the hippocampus. The alterations in the neuroinflammatory and neuroendocrine responses, together with the adaptive changes of synapse plasticity and Wnt signaling pathway, supported again the network hypothesis of AD that compromised functionality of relevant neural networks may underlie the development of AD symptomatology.

STZ belongs to the family of glucosamine-nitrosourea compound and DNA alkylating reagent, and could be transferred to the cells alone by means of glucose transporters protein 2 (GLUT2). Peripheral injection of STZ is used widely to induce animal models of diabetes mellitus, as it could kill the pancreatic β cells high selectively at a dose-dependently manner and induce glucose metabolism disorder. Given the important role of insulin in the regulation of synaptic plasticity and the close relationship between diabetes mellitus and AD, STZ is also reported to be used in establishing AD animal models (Agrawal et al., 2011; Rai et al., 2014). It has been reported that intracerebroventricular (icv) injection of STZ could cause not only the dysfunction of insulin/ insulin-like growth factor (IGF) pathway, but also the impaired learning and memory ability in mice (Agrawal et al., 2011). Consistently, in the present study, our results showed that bilateral injection of STZ into the dorsal hippocampus could induce a significant increase of serum insulin level in mice. These results suggest that there might other mechanism underling the effect of STZ on insulin secretion and glucose regulation, besides directly killing the pancreatic β cells.

To investigate the behavioral changes induced by central administration of STZ and its subsequent increase of serum insulin concentration, a series of behavioral tests were performed in the present study. The results showed that hippocampal injected with STZ could induce an impairment of learning and memory in mice, together with decreased spontaneous movement and exploratory behaviors, which was consistent with the findings in previous studies of STZ icv injected mice (Zhang et al., 2016; Sorial and El Sayed, 2017; Moreira-Silva et al., 2018). There was no significant difference between groups as regard to the behavioral performance in the TST, SPT, and RT, indicating that a bilaterally hippocampal injection of STZ might not enough to induce despair, anhedonia, and impairments of balance and coordination activity in mice. On the whole, these results showed that bilateral hippocampal STZ injection could successfully induce a turbulence of insulin abundance and trigger task-specific AD-like behavior changes in mice. Together with the increased expression Aβ_1−42_ and Tau in the hippocampus and PFC, our results indicated that bilaterally hippocampal injection of STZ should be an effective method to establish an AD-like mice model, with the advantages of clear location, accurate dose and direct effect.

Nesfatin-1 is a satiety factor widely expressed in both central and peripheral tissues. Apart from the function of regulating feeding behavior and energy metabolism, nesfatin-1 has been reported to participate in the pathogenesis of anxiety, depression and other neuropsychiatric behaviors. Results of our previous study demonstrated that peripheral abundance of nesfatin-1 could be changed by high-fat diet and chronic stress, and ip injection of nesfatin-1 could induce anxiety and depression-like behavioral disorder in rats, together with the change of hippocampal synaptic plasticity (Ge et al., 2015; Chen et al., 2019). Higher plasma level of nesfatin-1 was also reported in AD or diabetes mellitus patients and animal models (Dong et al., 2013; Alpua and Kisa, 2019), with significant correlation to the cognitive dysfunction. In the present study, our results showed that the serum nesfatin-1 concentration was increased in the bilaterally hippocampal STZ-injected mice. Although the mechanism remains unclear, these results provide new evidence that nesfatin-1 might be involved with the insulin metabolism disorder-associated cognitive impairment in AD.

Neuro-inflammation has long been taken as a critical factor in the pathological of AD. It has been demonstrated that microglia can be activated by Aβ deposition, thereby secreting various inflammatory cytokines and inducing the activation of the complement system (Marttinen et al., 2018). In turn, the reactive inflammatory response will induce a further activation of microglia, as a vicious circle, ultimately leading to the neuronal dysfunction. Iba-1 is a specific marker of microglia, and CD68 is also used to label activated microglia. Increased expressions of Iba-1 and GFAP have been identified in the hippocampus of adult rats or mice received icv administration of STZ (Dos Santos et al., 2020). In line with these findings, in the present study, the activation of microglia was observed in the STZ model group as indicated by aggregation and increased expression of Iba-1 and CD68 in the dentate gyrus of hippocampal tissues, The data is consistent with the phenomenon of a marked increase in CD68-labeled activated microglia in AD brain slices of APP/PS1 transgenic mouse (Gallagher et al., 2012). However, there was no significant difference between groups as regard to the expression of GFAP, which is taken as a marker of astrocytes activation. Although there was no exact interpret for this discrepancy, it has been reported that microglial cells act as first line of defense against immune responses in the brain, while astrocytes are neurosupportive in nature (Kraft et al., 2009; Kirkley et al., 2017). Microglial responses are often rapid, as compared with the more delayed activation of astrocytes against stimulation (Zhang et al., 2010; Kaur et al., 2019). Moreover, in many neurodegenerative diseases including AD, activation of microglia is reported to precede astrogliosis and overt neuronal loss (Kirkley et al., 2017). Although the dynamic changes of both microglia and astrocytes activation were not observed in this study, our results, together with the findings in other reports, suggested again that temporally distinct signaling events are required for a reactive phenotype in different type of glial cell.

The doublecortin (DCX) is considered as a molecular marker of neurogenesis in hippocampal dentate gyrus and only expressed in the newborn neurons, and significantly reduced number of newborn neurons labeled with DCX was demonstrated in the hippocampus of an AD rat model induced by icv injection of STZ (Mishra et al., 2018). In this present study, there was just a downward trend of DCX immune-positive cells in the dentate gyrus of the model mice, without a significant difference between groups. These results indicated that the reactive inflammatory response was activated after bilaterally hippocampal injection of STZ, together with subtle decline of neurogenesis. These changes might interpret partly the impairment of learning and memory of AD-like model mice induced by STZ hippocampal injection. However, more detailed investigation should be carried out focusing on the delicate shifts and the potential mechanism in the future.

Brain-derived neurotrophic factor (BDNF) can provide neurotrophic support for different neuronal populations including 5-HT and is a seminal regulator of synaptic plasticity. Decreased BDNF concentration has been reported in serum and CSF of AD patients, with a significantly positive relationship between the degree of decline and the severity of cognitive dysfunction (Li et al., 2009; Ng et al., 2019). Moreover, it has been demonstrated that lateral ventricle injection of nerve growth factor can significantly improve the cognitive abilities of AD transgenic mice by up-regulating the hippocampal expression of BDNF. Furthermore, BDNF can reverse neuron loss, improve cognitive impairment, and provide substantial protection for important neural circuits associated with AD (Nigam et al., 2017). Consistent with these findings, our results showed a decreased protein expression of BDNF in the hippocampus and the PFC of bilaterally hippocampal STZ-injected mice, suggesting the central role of BDNF in the pathogenesis of AD again. It has been reported that astrocytes could secrete BDNF to promote the development and survival of the central nervous system (de Pins et al., 2019). However, in the present study, the results of immunofluorescent staining showed no significant difference between groups about the number of astrocytes in the DG, indicating that the decreased expression of BDNF in the hippocampus and PFC of STZ-injected mice may not be regulated totally by the astrocytes.

NMDARs serve as modulators of synaptic transmission in the mammalian central nervous system with both short-term and long-lasting effects, and take responsible for maintaining the neuronal excitability, Ca^2+^ influx, and memory formation (Kamat et al., 2016). BDNF is reported to bind with TrkB and promote the activation of NMDAR and the aggregation of PSD95 through the PI3K-Akt pathway to regulate synaptic plasticity (Yoshii and Constantine-Paton, 2007). Over-activation of NMDAR could lead to neuronal cell death, excessive influx of Ca^2+^, and abnormal generation of free radicals, which drives synaptic dysfunction and hyper-phosphorylation of Tau protein (Rai et al., 2013). Memantine, the NMDA receptor antagonist, has been reported to improve the cognitive function of AD patients by improving the activity of glutamatergic neurons (Hong et al., 2018; Jason and Andrew, 2018). Besides, the content of phosphorylated and non-phosphorylated NMDAR subunits in the hippocampus of AD patients are reduced (Sze et al., 2001). Consistently, in SAMP8 mice or AD models injected with Aβ_25−35_ or Aβ_1−42_, the expression of NMDAR1, NMDAR2A, and NMDAR2B were decreased markedly in the hippocampus or cortex (Xu et al., 2016; Wang K. et al., 2018; Chang et al., 2020). Moreover, it has been demonstrated that ketamine, a non-competitive NMDAR antagonist, could ameliorate the impaired learning and memory of developing mice induced by repetitive mechanical stress, and the mechanism may be associated with the increased hippocampal BDNF expression (Peng et al., 2011; Chang et al., 2018). In this present study, the expression of NMDAR2A and NMDAR2B in the hippocampus and PFC of mice were significantly down-regulated after bilaterally hippocampal injection of STZ, and a significant positive correlation was found between the protein expression of BDNF and NMDAR2A. These results further confirmed the central role of BDNF in the pathogenesis of AD and the close relationship between BDNF and NMDARs.

Synaptic plasticity is the material basis for regulating learning and memory functions and emotional states. The PDZ domain of PSD95 can be combined with the intracellular domain of the NMDAR2 subunit, thereby anchoring NMDAR to the post-synaptic densities, based on which NMDAR can exert a biological function on synapses (Chen et al., 2015). Unsurprising, PSD95 is reported decreased in the hippocampus of APP/PS1 AD transgenic mice (Zhang et al., 2020). SynGAP, a protein enriched in excitatory synapses, can integrate with PSD95, Shank and Ca^2+^/CaMKII to form a complex that plays a key role in synaptic plasticity (Kim et al., 1998). Aβ oligomers have been reported to be capable of instigating synapse dysfunction and deterioration via binding with SynGAP, subsequently aggravating the course of AD (Ding et al., 2019). Results of postmortem study showed that the SynGAP levels in the PSD were remarkably declined in the PFC of AD patients (Gong et al., 2009). Compared with that of control group, the expressions of SynGAP and PSD95 in the hippocampus and the PFC of AD model mice was significantly down-regulated in the present study. Apart from the significant positive correlation between BDNF and NMDAR2A, the protein expression of SynGAP and PSD95 were also remarkably correlated with the expression of BDNF. Together with the shifted expression of BDNF and NMDARs in this study, our result suggests again that the synaptic plasticity, involved with the balance of multiple proteins and multiple receptors, play a critical role in the pathogenesis of AD.

Dysfunction of the Wnt/β-catenin signaling pathway is also involved in the progress of AD, which is manifested by the decreased level in β-catenin and increased activity of GSK-3β. The inhibition of Wnt/β-catenin pathway is reported to promote massive accumulation of Aβ and Tau hyper-phosphorylation, and Aβ in turn inhibits the Wnt/β-catenin pathway, eventually resulting in neurofibrillary tangles in AD patients and worsening the course of AD (Jia et al., 2019). Moreover, it has been demonstrated that blocking Wnt/β-catenin pathway could down-regulate the expression of BDNF induced by NMDAR activation in the primary cortical neurons of mice, whereas activation of Wnt/β-catenin pathway could stimulate and increase BDNF expression (Zhang et al., 2018). In this present study, the phosphorylation of β-catenin and GSK-3β (ser9) were decreased in the hippocampus or PFC of mice after bilaterally hippocampal injection of STZ, although the expression of CyclinD1 was not significantly different between groups. The phosphorylation of GSK-3β at ser9 inhibited the activity of GSK-3β, and the reduced phosphorylation of GSK-3β (ser9) in the hippocampus indicated the abnormal increased activity of GSK-3β. It has been demonstrated that GSK-3β could directly inhibit the expression of BDNF by phosphorylation of CREB (Grimes and Jope, 2001). In the present study, the protein expression of p-GSK-3β/GSK-3β was positively correlated with BDNF. These results indicated again the expression pattern of Wnt/β-catenin signaling pathway might be associated with the pathogenesis of STZ-induced AD, and BDNF might be taken as an important factor linking the imbalanced protein expression to the changed synaptic plasticity.

There were several limitations in the current study. First, although the central role of BDNF in the AD-like neurobiological changes induced by bilateral hippocampal STZ injection was suggested, it was not further identified by intervening the abundance or function of BDNF. Moreover, the correlation between BDNF and insulin or nesfatin-1, need to be observed in our further studies. Additionally, although increased expression of Aβ_1−42_ and Tau was demonstrated in this study, it is better to investigate the abundance of Aβ and more phosphorylation-site of Tau in detail.

In summary, our results showed that a bilaterally hippocampal injection of STZ could successfully induce AD-like behaviors in mice. Moreover, dysfunctional neuroendocrine response was also found in the bilaterally hippocampal STZ-injected mice, as indicated by the increased serum concentration of insulin and nesfatin-1. Apart from the hippocampal inflammatory response, the mechanism might be associated with the imbalanced expression of synaptic plasticity-related proteins in hippocampus and PFC, including NMDAR2A, NMDAR2B, SynGAP, PSD95, and BDNF. Moreover, the dysfunction of Wnt/β-catenin pathway, especially the decrease in the phosphorylation of β-catenin and GSK-3β, may also be involved in AD-like pathological process. Given the relationship between BDNF and the imbalanced expression of proteins associated with regulation of synaptic plasticity in the hippocampus and the PFC, the central role of BDNF was self-evident. These findings might provide new evidence for understanding the pathogenesis of STZ-associated AD.

## Data Availability Statement

The original contributions presented in the study are included in the article/Supplementary Material, further inquiries can be directed to the corresponding author.

## Ethics Statement

The animal study was reviewed and approved by the Animal Experimentation Ethics Committee of Tongji University School of Medicine.

## Author Contributions

C-CQ and X-XC performed most of the experiments, analyzed the data, and wrote the manuscript. X-RG performed Western blotting analysis. J-XX and SL modified the pictures. C-CQ and J-FG designed of the study. J-FG conceived the study and revised the manuscript. All authors read and approved the final manuscript.

## Conflict of Interest

The authors declare that the research was conducted in the absence of any commercial or financial relationships that could be construed as a potential conflict of interest.

## Abbreviations

Aβ: amyloid beta
AD: Alzheimer's disease
ADDLs: Aβ-derived diffusible ligands
CFC: contextual fear conditioning test
CREB: cAMP response element-binding protein
DCX: doublecortin
ELISA: enzyme-linked immunosorbent assay
FDA: American Food and Drug Administration
GFAP: glial fibrillary acidic protein
GLUT2: glucose transporters protein 2
Iba-1: ionized calcium-binding adapter molecule 1
IGF: insulin-like growth factor
MWM: Morris water maze
NFTs: nerve fiber tangles
NMDARs: N-methyl-D-aspartate receptor
NOR: novel object recognition test
OFT: open field test
PFC: pre-frontal cortex
PSD95: postsynaptic density protein 95
RT: rotarod test
SPs: senile plaques
SPI: sucrose preference index
SPT: sucrose preference test
STZ: streptozotocin
SynGAP: Synaptic Ras GTPase activation protein
TST: tail suspension test.

## References

[B1] Agrawal R. Tyagi E. Shukla R. Nath C. (2011). Insulin receptor signaling in rat hippocampus: a study in STZ (ICV) induced memory deficit model. Eur. Neuropsychopharmacol. 21, 261–273. 10.1016/j.euroneuro.2010.11.009 21195590

[B2] Alpua M. Kisa U. (2019). Nesfatin-1 and caspase-cleaved cytokeratin-18: promising biomarkers for Alzheimer's disease? Bratisl. Lek. Listy 120, 295–298. 10.4149/BLL_2019_046 31023053

[B3] Ballesteros J. J. Buschler A. Köhr G. Manahan-Vaughan D. (2016). Afferent input selects NMDA receptor subtype to determine the persistency of hippocampal LTP in freely behaving mice. Front. Synaptic Neurosci. 8:33. 10.3389/fnsyn.2016.00033 27818632 PMC5073893

[B4] Brigman J. L. Wright T. Talani G. Prasad-Mulcare S. Jinde S. Seabold G. K.. (2010). Loss of GluN2B-containing NMDA receptors in CA1 hippocampus and cortex impairs long-term depression, reduces dendritic spine density, and disrupts learning. J. Neurosci. 30, 4590–4600. 10.1523/JNEUROSCI.0640-10.2010 20357110 PMC2869199

[B5] Chang C. H. Su C. L. Gean P. W. (2018). Mechanism underlying NMDA blockade-induced inhibition of aggression in post-weaning socially isolated mice. Neuropharmacology 143, 95–105. 10.1016/j.neuropharm.2018.09.019 30243915

[B6] Chang K. W. Zong H. F. Rizvi M. Y. Ma K. G. Zhai W. Wang M.. (2020). Modulation of the MAPKs pathways affects Abeta-induced cognitive deficits in Alzheimer's disease via activation of alpha7nAChR. Neurobiol. Learn. Mem. 168:107154. 10.1016/j.nlm.2019.107154 31904546

[B7] Chen X. Levy J. M. Hou A. Winters C. Azzam R. Sousa A. A.. (2015). PSD-95 family MAGUKs are essential for anchoring AMPA and NMDA receptor complexes at the postsynaptic density. Proc. Natl. Acad. Sci. U. S. A. 112, E6983–6992. 10.1073/pnas.1517045112 26604311 PMC4687590

[B8] Chen X.-X. Xu Y.-Y. Wu R. Chen Z. Fang K. Han Y.-X.. (2019). Resveratrol reduces glucolipid metabolic dysfunction and learning and memory impairment in a NAFLD rat model: involvement in regulating the imbalance of Nesfatin-1 abundance and Copine 6 expression. Front. Endocrinol. (Lausanne) 10:434. 10.3389/fendo.2019.00434 31338065 PMC6629830

[B9] Dai J. X. Han H. L. Tian M. Cao J. Xiu J. B. Song N. N.. (2008). Enhanced contextual fear memory in central serotonin-deficient mice. Proc. Natl. Acad. Sci. U. S. A. 105, 11981–11986. 10.1073/pnas.0801329105 18695238 PMC2575315

[B10] de Pins B. Cifuentes-Diaz C. Farah A. T. Lopez-Molina L. Montalban E. Sancho-Balsells A.. (2019). Conditional BDNF delivery from astrocytes rescues memory deficits, spine density, and synaptic properties in the 5xFAD mouse model of Alzheimer disease. J. Neurosci. 39, 2441–2458. 10.1523/JNEUROSCI.2121-18.2019 30700530 PMC6435824

[B11] Devi L. Ohno M. (2013). Effects of levetiracetam, an antiepileptic drug, on memory impairments associated with aging and Alzheimer's disease in mice. Neurobiol. Learn. Mem. 102, 7–11. 10.1016/j.nlm.2013.02.001 23416036

[B12] Ding Y. Zhao J. Zhang X. Wang S. Viola K. L. Chow F. E.. (2019). Amyloid beta oligomers target to extracellular and intracellular neuronal synaptic proteins in Alzheimer's disease. Front. Neurol. 10:1140. 10.3389/fneur.2019.01140 31736856 PMC6838211

[B13] Dong J. Xu H. Xu H. Wang P. F. Cai G. J. Song H. F.. (2013). Nesfatin-1 stimulates fatty-acid oxidation by activating AMP-activated protein kinase in STZ-induced type 2 diabetic mice. PLoS ONE 8:e83397. 10.1371/journal.pone.0083397 24391760 PMC3877039

[B14] Dos Santos J. P. A. Vizuete A. F. Goncalves C. A. (2020). Calcineurin-mediated hippocampal inflammatory alterations in streptozotocin-induced model of dementia. Mol. Neurobiol. 57, 502–512. 10.1007/s12035-019-01718-2 31388960

[B15] Folke J. Pakkenberg B. Brudek T. (2019). Impaired Wnt signaling in the prefrontal cortex of Alzheimer's disease. Mol. Neurobiol. 56, 873–891. 10.1007/s12035-018-1103-z 29804228

[B16] Gaesser J. M. Fyffe-Maricich S. L. (2016). Intracellular signaling pathway regulation of myelination and remyelination in the CNS. Exp. Neurol. 283, 501–511. 10.1016/j.expneurol.2016.03.008 26957369 PMC5010983

[B17] Gallagher J. J. Finnegan M. E. Grehan B. Dobson J. Collingwood J. F. Lynch M. A. (2012). Modest amyloid deposition is associated with iron dysregulation, microglial activation, and oxidative stress. J. Alzheimers. Dis. 28, 147–161. 10.3233/JAD-2011-110614 21971404

[B18] Ge J.-F. Xu Y.-Y. Qin G. Peng Y.-N. Zhang C.-F. Liu X.-R.. (2015). Depression-like behavior induced by Nesfatin-1 in rats: involvement of increased immune activation and imbalance of synaptic vesicle proteins. Front. Neurosci. 9:429. 10.3389/fnins.2015.00429 26617482 PMC4639614

[B19] Gong Y. Lippa C. F. Zhu J. Lin Q. Rosso A. L. (2009). Disruption of glutamate receptors at Shank-postsynaptic platform in Alzheimer's disease. Brain Res. 1292, 191–198. 10.1016/j.brainres.2009.07.056 19635471 PMC2745956

[B20] Grimes C. A. Jope R. S. (2001). CREB DNA binding activity is inhibited by glycogen synthase kinase-3 beta and facilitated by lithium. J. Neurochem. 78, 1219–1232. 10.1046/j.1471-4159.2001.00495.x 11579131 PMC1947002

[B21] Henneberger C. Bard L. King C. Jennings A. Rusakov D. A. (2013). NMDA receptor activation: two targets for two co-agonists. Neurochem. Res. 38, 1156–1162. 10.1007/s11064-013-0987-2 23389661

[B22] Hiser J. Koenigs M. (2018). The multifaceted role of the ventromedial prefrontal cortex in emotion, decision making, social cognition, and psychopathology. Biol. Psychiatry 83, 638–647. 10.1016/j.biopsych.2017.10.030 29275839 PMC5862740

[B23] Hong Y. J. Choi S. H. Jeong J. H. Park K. W. Na H. R. (2018). Effectiveness of anti-dementia drugs in extremely severe Alzheimer's disease: a 12-week, multicenter, randomized, single-blind study. J. Alzheimers. Dis. 63, 1035–1044. 10.3233/JAD-180159 29710726

[B24] Hung S. Y. Fu W. M. (2017). Drug candidates in clinical trials for Alzheimer's disease. J. Biomed. Sci. 24:47. 10.1186/s12929-017-0355-7 28720101 PMC5516350

[B25] Jahanshahi M. Saeidi M. Nikmahzar E. Babakordi F. Bahlakeh G. (2019). Effects of hCG on reduced numbers of hCG receptors in the prefrontal cortex and cerebellum of rat models of Alzheimer's disease. Biotech. Histochem. 94, 360–365. 10.1080/10520295.2019.1571228 30760053

[B26] Jason W. Andrew B. (2018). Current understanding of Alzheimer's disease diagnosis and treatment. F1000Research 7:1. 10.12688/f1000research.14506.1 30135715 PMC6073093

[B27] Jia L. Pina-Crespo J. Li Y. (2019). Restoring Wnt/beta-catenin signaling is a promising therapeutic strategy for Alzheimer's disease. Mol. Brain 12:104. 10.1186/s13041-019-0525-5 31801553 PMC6894260

[B28] Kamat P. K. Kalani A. Rai S. Swarnkar S. Tota S. Nath C.. (2016). Mechanism of oxidative stress and synapse dysfunction in the pathogenesis of Alzheimer's disease: understanding the therapeutics strategies. Mol. Neurobiol. 53, 648–661. 10.1007/s12035-014-9053-6 25511446 PMC4470891

[B29] Kandimalla R. Manczak M. Yin X. Wang R. Reddy P. H. (2018). Hippocampal phosphorylated tau induced cognitive decline, dendritic spine loss and mitochondrial abnormalities in a mouse model of Alzheimer's disease. Hum. Mol. Genet. 27, 30–40. 10.1093/hmg/ddx381 29040533 PMC5886218

[B30] Kaur D. Sharma V. Deshmukh R. (2019). Activation of microglia and astrocytes: a roadway to neuroinflammation and Alzheimer's disease. Inflammopharmacology 27, 663–677. 10.1007/s10787-019-00580-x 30874945

[B31] Ke F. Li H.-R. Chen X.-X. Gao X.-R. Huang L.-L. Du A.-Q.. (2020). Quercetin alleviates LPS-induced depression-like behavior in rats via regulating BDNF-related imbalance of copine 6 and TREM1/2 in the hippocampus and PFC. Front. Pharmacol. 10:1544. 10.3389/fphar.2019.01544 32009956 PMC6978986

[B32] Kim J. H. Liao D. Lau L. F. Huganir R. L. (1998). SynGAP: a synaptic RasGAP that associates with the PSD-95/SAP90 protein family. Neuron 20, 683–691. 10.1016/S0896-6273(00)81008-9 9581761

[B33] Kirkley K. S. Popichak K. A. Afzali M. F. Legare M. E. Tjalkens R. B. (2017). Microglia amplify inflammatory activation of astrocytes in manganese neurotoxicity. J. Neuroinflammation. 14:99. 10.1186/s12974-017-0871-0 28476157 PMC5418760

[B34] Kraft A. D. McPherson C. A. Harry G. J. (2009). Heterogeneity of microglia and TNF signaling as determinants for neuronal death or survival. Neurotoxicology 30, 785–793. 10.1016/j.neuro.2009.07.001 19596372 PMC3329780

[B35] Li G. Peskind E. R. Millard S. P. Chi P. Sokal I. Yu C. E.. (2009). Cerebrospinal fluid concentration of brain-derived neurotrophic factor and cognitive function in non-demented subjects. PLoS ONE 4:e5424. 10.1371/journal.pone.0005424 19412541 PMC2671606

[B36] Li H. Wu J. Zhu L. Sha L. Yang S. Wei J.. (2018). Insulin degrading enzyme contributes to the pathology in a mixed model of Type 2 diabetes and Alzheimer's disease: possible mechanisms of IDE in T2D and AD. Biosci. Rep. 38:BSR20170862. 10.1042/BSR20170862 29222348 PMC6435468

[B37] Liu C. C. Tsai C. W. Deak F. Rogers J. Penuliar M. Sung Y. M.. (2014). Deficiency in LRP6-mediated Wnt signaling contributes to synaptic abnormalities and amyloid pathology in Alzheimer's disease. Neuron 84, 63–77. 10.1016/j.neuron.2014.08.048 25242217 PMC4199382

[B38] Magdesian M. H. Carvalho M. M. Mendes F. A. Saraiva L. M. Juliano M. A. Juliano L.. (2008). Amyloid-beta binds to the extracellular cysteine-rich domain of Frizzled and inhibits Wnt/beta-catenin signaling. J. Biol. Chem. 283, 9359–9368. 10.1074/jbc.M707108200 18234671 PMC2431018

[B39] Marttinen M. Takalo M. Natunen T. Wittrahm R. Gabbouj S. Kemppainen S.. (2018). Molecular mechanisms of synaptotoxicity and neuroinflammation in Alzheimer's disease. Front. Neurosci. 12:963. 10.3389/fnins.2018.00963 30618585 PMC6301995

[B40] Miao Y. Wang N. Shao W. Xu Z. Yang Z. Wang L.. (2019). Overexpression of TIPE2, a negative regulator of innate and adaptive immunity, attenuates cognitive deficits in APP/PS1 mice. J. Neuroimmune Pharmacol. 14, 519–529. 10.1007/s11481-019-09861-2 31286344

[B41] Mishra S. K. Singh S. Shukla S. Shukla R. (2018). Intracerebroventricular streptozotocin impairs adult neurogenesis and cognitive functions via regulating neuroinflammation and insulin signaling in adult rats. Neurochem. Int. 113, 56–68. 10.1016/j.neuint.2017.11.012 29174383

[B42] Moreira-Silva D. Carrettiero D. C. Oliveira A. S. A. Rodrigues S. Dos Santos-Lopes J. Canas P. M.. (2018). Anandamide effects in a streptozotocin-induced Alzheimer's disease-like sporadic dementia in rats. Front. Neurosci. 12:653. 10.3389/fnins.2018.00653 30333717 PMC6176656

[B43] Muller M. K. Jacobi E. Sakimura K. Malinow R. von Engelhardt J. (2018). NMDA receptors mediate synaptic depression, but not spine loss in the dentate gyrus of adult amyloid Beta (Abeta) overexpressing mice. Acta Neuropathol Commun. 6:110. 10.1186/s40478-018-0611-4 30352630 PMC6198500

[B44] Ng T. K. S. Ho C. S. H. Tam W. W. S. Kua E. H. Ho R. C. (2019). Decreased serum brain-derived neurotrophic factor (BDNF) levels in patients with Alzheimer's Disease (AD): a systematic review and meta-analysis. Int. J. Mol. Sci. 20:57. 10.3390/ijms20020257 30634650 PMC6358753

[B45] Nigam S. M. Xu S. Kritikou J. S. Marosi K. Brodin L. Mattson M. P. (2017). Exercise and BDNF reduce Abeta production by enhancing alpha-secretase processing of APP. J. Neurochem. 142, 286–296. 10.1111/jnc.14034 28382744 PMC5498234

[B46] Peng S. Zhang Y. Wang H. Ren B. Zhang J. (2011). Anesthetic ketamine counteracts repetitive mechanical stress-induced learning and memory impairment in developing mice. Mol. Biol. Rep. 38, 4347–4351. 10.1007/s11033-010-0561-9 21116850

[B47] Pruski M. Hu L. Yang C. Wang Y. Zhang J. B. Zhang L.. (2019). Roles for IFT172 and primary cilia in cell migration, cell division, and neocortex development. Front Cell Dev Biol. 7:287. 10.3389/fcell.2019.00287 31850339 PMC6890611

[B48] Rai S. Kamat P. K. Nath C. Shukla R. (2013). A study on neuroinflammation and NMDA receptor function in STZ (ICV) induced memory impaired rats. J. Neuroimmunol. 254, 1–9. 10.1016/j.jneuroim.2012.08.008 23021418

[B49] Rai S. Kamat P. K. Nath C. Shukla R. (2014). Glial activation and post-synaptic neurotoxicity: the key events in Streptozotocin (ICV) induced memory impairment in rats. Pharmacol. Biochem. Behav. 117, 104–117. 10.1016/j.pbb.2013.11.035 24333387

[B50] Rodriguez J. J. Noristani H. N. Olabarria M. Fletcher J. Somerville T. D. Yeh C. Y.. (2011). Voluntary running and environmental enrichment restores impaired hippocampal neurogenesis in a triple transgenic mouse model of Alzheimer's disease. Curr. Alzheimer Res. 8, 707–717. 10.2174/156720511797633214 21453244

[B51] Sorial M. E. El Sayed N. (2017). Protective effect of valproic acid in streptozotocin-induced sporadic Alzheimer's disease mouse model: possible involvement of the cholinergic system. Naunyn Schmiedebergs. Arch. Pharmacol. 390, 581–593. 10.1007/s00210-017-1357-4 28188358

[B52] Syed Y. Y. (2020). Correction to: Sodium oligomannate: first approval. Drugs 80, 445–446. 10.1007/s40265-020-01274-3 32096019

[B53] Sze C.-I. Bi H. Kleinschmidt-DeMasters B. K. Filley C. M. Martin L. J. (2001). N -Methyl- d -aspartate receptor subunit proteins and their phosphorylation status are altered selectively in Alzheimer's disease. J. Neurol. Sci. 182, 151–159. 10.1016/S0022-510X(00)00467-6 11137521

[B54] Tapia-Rojas C. Inestrosa N. C. (2018). Loss of canonical Wnt signaling is involved in the pathogenesis of Alzheimer's disease. Neural Regen. Res. 13, 1705–1710. 10.4103/1673-5374.238606 30136680 PMC6128062

[B55] Teixeira C. M. Pallas-Bazarra N. Bolos M. Terreros-Roncal J. Avila J. Llorens-Martin M. (2018). Untold New beginnings: adult hippocampal neurogenesis and Alzheimer's disease. J. Alzheimers Dis. 64, 497–505. 10.3233/JAD-179918 29562522

[B56] Wang C.-C. Held R. G. Chang S.-C. Yang L. Delpire E. Ghosh A.. (2011). A critical role for GluN2B-containing NMDA receptors in cortical development and function. Neuron 72, 789–805. 10.1016/j.neuron.2011.09.023 22153375

[B57] Wang D. Wang C. Liu L. Li S. (2018). Protective effects of evodiamine in experimental paradigm of Alzheimer's disease. Cogn. Neurodyn. 12, 303–313. 10.1007/s11571-017-9471-z 29765479 PMC5943208

[B58] Wang K. Sun W. Zhang L. Guo W. Xu J. Liu S.. (2018). Oleanolic acid ameliorates abeta25-35 injection-induced memory deficit in Alzheimer's disease model rats by maintaining synaptic plasticity. CNS Neurol. Disord. Drug Targets 17, 389–399. 10.2174/1871527317666180525113109 29793416 PMC6327117

[B59] Wang X. Sun G. Feng T. Zhang J. Huang X. Wang T.. (2019). Sodium oligomannate therapeutically remodels gut microbiota and suppresses gut bacterial amino acids-shaped neuroinflammation to inhibit Alzheimer's disease progression. Cell Res. 29, 787–803. 10.1038/s41422-019-0216-x 31488882 PMC6796854

[B60] Xu P. Xu S. P. Wang K. Z. Lu C. Zhang H. X. Pan R. L.. (2016). Cognitive-enhancing effects of hydrolysate of polygalasaponin in SAMP8 mice. J. Zhejiang Univ. Sci. B 17, 503–514. 10.1631/jzus.B1500321 27381727 PMC4940626

[B61] Yoshii A. Constantine-Paton M. (2007). BDNF induces transport of PSD-95 to dendrites through PI3K-AKT signaling after NMDA receptor activation. Nat. Neurosci. 10, 702–711. 10.1038/nn1903 17515902

[B62] Zameer S. Kaundal M. Vohora D. Ali J. Kalam Najmi A. Akhtar M. (2019). Ameliorative effect of alendronate against intracerebroventricular streptozotocin induced alteration in neurobehavioral, neuroinflammation and biochemical parameters with emphasis on Abeta and BACE-1. Neurotoxicology 70, 122–134. 10.1016/j.neuro.2018.11.012 30481507

[B63] Zhang C. T. Lin J. R. Wu F. Ghosh A. Tang S. S. Hu M.. (2016). Montelukast ameliorates streptozotocin-induced cognitive impairment and neurotoxicity in mice. Neurotoxicology 57, 214–222. 10.1016/j.neuro.2016.09.022 27702591

[B64] Zhang D. Hu X. Qian L. O'Callaghan J. P. Hong J. S. (2010). Astrogliosis in CNS pathologies: is there a role for microglia? Mol. Neurobiol. 41, 232–241. 10.1007/s12035-010-8098-4 20148316 PMC3629545

[B65] Zhang Q. Huang Y. Zhang L. Ding Y. Q. Song N. N. (2019). Loss of Satb2 in the cortex and hippocampus leads to abnormal behaviors in mice. Front. Mol. Neurosci. 12:33. 10.3389/fnmol.2019.00033 30809123 PMC6380165

[B66] Zhang W. Shi Y. Peng Y. Zhong L. Zhu S. Zhang W.. (2018). Neuron activity-induced Wnt signaling up-regulates expression of brain-derived neurotrophic factor in the pain neural circuit. J. Biol. Chem. 293, 15641–15651. 10.1074/jbc.RA118.002840 30139740 PMC6177598

[B67] Zhang X. Zhao F. Wang C. Zhang J. Bai Y. Zhou F.. (2020). AVP(4-8) improves cognitive behaviors and hippocampal synaptic plasticity in the APP/PS1 Mouse model of Alzheimer's disease. Neurosci. Bull. 36, 254–262. 10.1007/s12264-019-00434-0 31605298 PMC7056786

[B68] Zhao Y. Kiss T. DelFavero J. Li L. Li X. Zheng L.. (2020). CD82-TRPM7-Numb signaling mediates age-related cognitive impairment. Geroscience 42, 595–611. 10.1007/s11357-020-00166-4 32088828 PMC7205934

[B69] Zhong S. Zhang S. Fan X. Wu Q. Yan L. Dong J.. (2018). A single-cell RNA-seq survey of the developmental landscape of the human prefrontal cortex. Nature 555, 524–528. 10.1038/nature25980 29539641

[B70] Zhou Y. Wang X. Zhao Y. Liu A. Zhao T. Zhang Y.. (2016). Elevated thyroid peroxidase antibody increases risk of post-partum depression by decreasing prefrontal cortex BDNF and 5-HT levels in mice. Front. Cell. Neurosci. 10:307. 10.3389/fncel.2016.00307 28119573 PMC5220058

[B71] Zhu F. Zheng Y. Ding Y. Q. Liu Y. Zhang X. Wu R.. (2014). Minocycline and risperidone prevent microglia activation and rescue behavioral deficits induced by neonatal intrahippocampal injection of lipopolysaccharide in rats. PLoS ONE 9:e93966. 10.1371/journal.pone.0093966 24705495 PMC3976322

